# REDISCOVER International Guidelines on the Perioperative Care of Surgical Patients With Borderline-resectable and Locally Advanced Pancreatic Cancer

**DOI:** 10.1097/SLA.0000000000006248

**Published:** 2024-02-26

**Authors:** Ugo Boggi, Emanuele Kauffmann, Niccolò Napoli, S. George Barreto, Marc G. Besselink, Giuseppe K. Fusai, Thilo Hackert, Mohammad Abu Hilal, Giovanni Marchegiani, Roberto Salvia, Shailesh V. Shrikhande, Mark Truty, Jens Werner, Christopher L. Wolfgang, Elisa Bannone, Giovanni Capretti, Alice Cattelani, Alessandro Coppola, Alessandro Cucchetti, Davide De Sio, Armando Di Dato, Giovanna Di Meo, Claudio Fiorillo, Cesare Gianfaldoni, Michael Ginesini, Camila Hidalgo Salinas, Quirino Lai, Mario Miccoli, Roberto Montorsi, Michele Pagnanelli, Andrea Poli, Claudio Ricci, Francesco Sucameli, Domenico Tamburrino, Virginia Viti, Pietro F. Addeo, Sergio Alfieri, Philippe Bachellier, Gian Luca Baiocchi, Gianpaolo Balzano, Linda Barbarello, Alberto Brolese, Juli Busquets, Giovanni Butturini, Fabio Caniglia, Damiano Caputo, Riccardo Casadei, Xi Chunhua, Ettore Colangelo, Andrea Coratti, Francesca Costa, Francesco Crafa, Raffaele Dalla Valle, Luciano De Carlis, Roeland F. de Wilde, Marco Del Chiaro, Fabrizio Di Benedetto, Pierluigi Di Sebastiano, Safi Dokmak, Melissa Hogg, Vyacheslav I. Egorov, Giorgio Ercolani, Giuseppe Maria Ettorre, Massimo Falconi, Giovanni Ferrari, Alessandro Ferrero, Marco Filauro, Alessandro Giardino, Gian Luca Grazi, Salvatore Gruttadauria, Jakob R Izbicki, Elio Jovine, Matthew Katz, Tobias Keck, Igor Khatkov, Gozo Kiguchi, David Kooby, Hauke Lang, Carlo Lombardo, Giuseppe Malleo, Marco Massani, Vincenzo Mazzaferro, Riccardo Memeo, Yi Miao, Kohei Mishima, Carlo Molino, Yuichi Nagakawa, Masafumi Nakamura, Bruno Nardo, Fabrizio Panaro, Claudio Pasquali, Vittorio Perrone, Elena Rangelova, Rong Liu, Renato Romagnoli, Raffaele Romito, Edoardo Rosso, Richard Schulick, Ajith Siriwardena, Marcello Giuseppe Spampinato, Oliver Strobel, Mario Testini, Roberto Ivan Troisi, Faik G. Uzunoglo, Roberto Valente, Luigi Veneroni, Alessandro Zerbi, Emilio Vicente, Fabio Vistoli, Marco Vivarelli, Go Wakabayashi, Giacomo Zanus, Amer Zureikat, Nicholas J. Zyromski, Roberto Coppola, Vito D’Andrea, José Davide, Christos Dervenis, Isabella Frigerio, Kevin C. Konlon, Fabrizio Michelassi, Marco Montorsi, William Nealon, Nazario Portolani, Donzília Sousa Silva, Giuseppe Bozzi, Viviana Ferrari, Maria G. Trivella, John Cameron, Pierre-Alain Clavien, Horacio J. Asbun, The REDISCOVER Multidisciplinary Advisory Board

**Affiliations:** * Division of General and Transplant Surgery, University of Pisa, Pisa, Italy; † College of Medicine and Public Health, Flinders University, Bedford Park, SA, Australia; ‡ Division of Surgery and Perioperative Medicine, Flinders Medical Center, Bedford Park, SA, Australia; § Department of Surgery, Amsterdam UMC, University of Amsterdam, Amsterdam, The Netherlands; ∥ Cancer Center Amsterdam, Amsterdam, The Netherlands; ¶ HPB & Liver Transplant Unit, Royal Free Hospital, London, UK; # Department of General, Visceral and Thoracic Surgery, University Hospital Hamburg-Eppendorf, Hamburg, Germany; ** Department of Surgery, Poliambulanza Foundation Hospital, Brescia, Italy; †† Hepatopancreatobiliary and Liver Transplant Surgery, Department of Surgery, Oncology and Gastroenterology, DiSCOG, University of Padua, Padua, Italy; ‡‡ General and Pancreatic Surgery Unit, Pancreas Institute, University of Verona, Verona, Italy; §§ Tata Memorial Hospital, Gastrointestinal and HPB Service, Homi Bhabha National Institute, Tata Memorial Centre, Mumbai, Maharashtra, India; ∥∥ Department of Surgery, Division of Hepatobiliary & Pancreas Surgery, Mayo Clinic Rochester, Rochester, MN; ¶¶ Department of General, Visceral, and Transplant Surgery, LMU, University of Munich, Munich, Germany; ## Department of Surgery, The NYU Grossman School of Medicine and NYU Langone Health, New York, NY; *** IRCCS Humanitas Research Hospital, Rozzano, Milan, Italy; ††† Department of Surgery, Sapienza University of Rome, Rome, Italy; ‡‡‡ Department of Medical and Surgical Sciences-DIMEC, Alma Mater Studiorum Università di Bologna, Bologna, Italy; §§§ Gemelli Pancreatic Center, CRMPG (Advanced Pancreatic Research Center), Fondazione Policlinico Universitario “Agostino Gemelli” IRCCS, UNIVERSITA' CATTOLICA DEL SACRO CUORE, Rome, Italy; ∥∥∥ Department of Precision and Regenerative Medicine and Ionian Area (DiMePre-J), University of Bari, Bari, Italy; ¶¶¶ Kellogg College, University of Oxford, Oxford, UK; ### Department of General and Specialty Surgery, Sapienza University of Rome, AOU Policlinico Umberto I of Rome, Rome, Italy; **** Department of Clinical and Experimental Medicine, University of Pisa, Pisa, Italy; †††† Department of Internal Medicine and Surgery (DIMEC), Alma Mater Studiorum, University of Bologna, Bologna, Italy; ‡‡‡‡ Division of Pancreatic Surgery, IRCCS, Azienda Ospedaliero-Universitaria di Bologna (IRCCS AOUBO), Bologna, Italy; §§§§ Division of Pancreatic Surgery, Pancreas Translational and Clinical Research Center, IRCCS San Raffaele Scientific Institute, Vita-Salute University, Milan, Italy; ∥∥∥∥ Division of Hepato-Pancreato-Biliary Surgery and Liver Transplantation, Hôpital de Hautepierre-Hôpitaux Universitaires de Strasbourg, Université de Strasbourg, Strasbourg, France; ¶¶¶¶ Department of Clinical and Experimental Sciences, University of Brescia and UOC General Surgery, ASST Cremona, Cremona, Italy; #### Department of General Surgery & HPB Unit, APSS, Trento, Italy; ***** Division of Pancreatobiliary Surgery and Liver Transplantation, Department of Surgery, Bellvitge University Hospital, IDIBELL, L´Hospitalet de Llobregat, Barcelona, Spain; ††††† Hepatopancreatobiliary Surgery, Pederzoli Hospital, Peschiera del Garda, Verona, Italy; ‡‡‡‡‡ Research Unit of General Surgery, Department of Medicine and Surgery, University Campus Bio-Medico di Roma, Rome, Italy; §§§§§ Operative Research Unit of General Surgery, Fondazione Policlinico Universitario Campus Bio-Medico, Roma, Italy; ∥∥∥∥∥ Pancreas Center, The First Affiliated Hospital of Nanjing Medical University, Nanjing, Jiangsu Province, People’s Republic of China; ¶¶¶¶¶ Pancreas Institute, Nanjing Medical University, Nanjing, Jiangsu Province, People’s Republic of China; ##### Department of General Surgery, The First Affiliated Hospital of Nanjing Medical University, Nanjing, Jiangsu Province, People’s Republic of China; ****** Department of General Surgery, “G. Mazzini” Hospital, Teramo, Italy; †††††† Department of General and Emergency Surgery, AUSL Toscana Sud Est, Misericordia Hospital of Grosseto, Grosseto, Italy; ‡‡‡‡‡‡ Division of General, Oncological and Robotic Surgery, San Giuseppe Moscati Hospital, Avellino, Italy; §§§§§§ Department of Medicine and Surgery, HPB Unit, University of Parma, Parma, Italy; ∥∥∥∥∥∥ Division of HPB Surgery and Transplantation, Niguarda Hospital, University of Milano-Bicocca, Milan, Italy; ¶¶¶¶¶¶ Department of Surgery, Erasmus MC Cancer Institute, Erasmus University Medical Center, Rotterdam, The Netherlands; ###### Department of Surgery, University of Colorado School of Medicine. Aurora, CO; ******* Hepato-Pancreato-Biliary Surgery and Liver Transplantation Unit, University of Modena and Reggio Emilia, Modena, Italy; ††††††† Surgical Oncology, Pierangeli Clinic, Department of Innovative Technology in Medicine & Dentistry, G. D’Annunzio University Chieti-Pescara, Chieti, Italy; ‡‡‡‡‡‡‡ Department of HPB Surgery and Liver Transplantation, Beaujon Hospital, Clichy, France; §§§§§§§ University Paris Cité, Paris, France; ∥∥∥∥∥∥∥ Department of Surgery, Division of HPB Surgery, NorthShore University HealthSystem, Evanston, IL; ¶¶¶¶¶¶¶ Department for Surgical Oncology and HPB Surgery, Ilyinskaya Hospital, Moscow, Russia; ####### Department of General Surgery and Transplantation. San Camillo Forlanini Hospital-POIT, Rome, Italy; ******** Division of Minimally-Invasive Surgical Oncology, ASST Grande Ospedale Metropolitano Niguarda, Milan, Italy; †††††††† Department of General and Oncological Surgery, “Umberto I” Mauriziano Hospital, Turin, Italy; ‡‡‡‡‡‡‡‡ Department of Surgery Galliera Hospital, Genova, Italy; §§§§§§§§ Department of Experimental and Clinical Medicine, Division of HepatoBiliaryPancreatic Surgery, AOU Careggi, University of Florence, Florence, Italy; ∥∥∥∥∥∥∥∥ Department for the Treatment and Study of Abdominal Diseases and Abdominal Transplantation, Istituto di Ricovero e Cura a Carattere Scientifico-Istituto Mediterraneo per i Trapianti e Terapie ad Alta Specializzazione (IRCCS-ISMETT), University of Pittsburgh Medical Center Italy (UPMC Italy), Palermo, Italy; ¶¶¶¶¶¶¶¶ Department of General Surgery and Medical-Surgical Specialties, University of Catania, Catania, Italy; ######## Department of General Visceral and Thoracic Surgery, University Hospital Eppendorf University of Hamburg, Hamburg, Germany; ********* Alma Mater Studiorum University of Bologna, IRCCS AOU of Bologna, Bologna, Italy; ††††††††† The University of Texas MD Anderson Cancer Center, Houston, TX; ‡‡‡‡‡‡‡‡‡ Department of Surgery, University Medical Center Schleswig-Holstein, Campus Lübeck, Lübeck, Germany; §§§§§§§§§ Department of High Technology Surgery, Moscow Clinical Scientific Center, Moscow, Russia; ∥∥∥∥∥∥∥∥∥ Department of Surgery, Hirakata Kohsai Hospital, Osaka, Japan; ¶¶¶¶¶¶¶¶¶ Department of Surgery, Emory University School of Medicine, Atlanta, GA; ######### University Medical Centre of the Johannes Gutenberg University Mainz, Mainz, Germany; ********** Department of Surgery, Regional Hospital of Treviso, Treviso, Italy; †††††††††† Department of Oncology and Hemato-Oncology, University of Milan HPB Surgery and Liver Transplantation Fondazione IRCCS Istituto Nazionale Tumori, Milan, Italy; ‡‡‡‡‡‡‡‡‡‡ Department of Hepato-Pancreatc-Biliary Surgery, “F. Miulli” General Regional Hospital, Acquaviva delle Fonti, Bari, Italy. Department of Medicine and Surgery, LUM University, Casamassima, Bari, Italy; §§§§§§§§§§ Pancreas Center, The Affiliated BenQ Hospital of Nanjing Medical University, Nanjing, Jiangsu Province, People’s Republic of China; ∥∥∥∥∥∥∥∥∥∥ Research Institute Against Digestive Cancer (IRCAD), Strasbourg, France; ¶¶¶¶¶¶¶¶¶¶ Department of General and Speciality Surgery, General and Pancreatic Surgery Team 1, AORN A. Cardarelli, Naples, Italy; ########## Department of Gastrointestinal and Pediatric Surgery, Tokyo Medical University, Tokyo, Japan; *********** Department of Surgery and Oncology, Graduate School of Medical Sciences, Kyushu University, Fukuoka, Japan; ††††††††††† Department of Surgery and Robotic, Division of General Surgery, Annunziata Hub Hospital, School of Medicine Surgery and TD, University of Calabria, Cosenza, Italy; ‡‡‡‡‡‡‡‡‡‡‡ Department of Surgery, Division of HBP Surgery & Transplantation, Montpellier University Hospital School of Medicine, Montpellier, France; §§§§§§§§§§§ Pancreatic & Digestive Endocrine Surgery Research Group—Department of Surgery, Oncology and Gastroenterology, DiSCOG, University of Padua, Padua, Italy; ∥∥∥∥∥∥∥∥∥∥∥ Section for Upper Abdominal Surgery at the Department of Surgery, Sahlgrenska University Hospital, Gothenburg, Sweden; ¶¶¶¶¶¶¶¶¶¶¶ Department of Surgery at the Institute of Clinical Sciences, Sahlgrenska Academy, University of Gothenburg, Gothenburg, Sweden; ########### Second Department of Hepatopancreatobiliary Surgery, Chinese People’s Liberation Army (PLA) General Hospital, Beijing, People’s Republic of China; ************ Division of General Surgery 2U-Liver Transplant Unit, Azienda Ospedaliero Universitaria Città della Salute e della Scienza di Torino, University of Turin, Turin, Italy; †††††††††††† Division of General Surgery II and HPB Unit, A.O.U. Maggiore della Carità di Novara, Novara, Italy; ‡‡‡‡‡‡‡‡‡‡‡‡ Service de Chirurgie Générale, Mini-Invasive et Robotique, Centre Hôspitalier de; §§§§§§§§§§§§ Regional Hepato-Pancreato-Biliary Unit, Manchester Royal Infirmary, Manchester, UK; ∥∥∥∥∥∥∥∥∥∥∥∥ Department of General and Minimally Invasive Surgery, “Vito Fazzi” Hospital, Lecce, Italy; ¶¶¶¶¶¶¶¶¶¶¶¶ Department of General Surgery, Division of Visceral Surgery, Medical University of Vienna, Vienna, Austria; ############ Division of HBP, Minimally Invasive and Robotic Surgery, Transplantation Service Federico II University Hospital, Naples, Italy; ************* Department of Surgery, ASL3 Genovese, Genoa, Italy; ††††††††††††† Chirurgia Generale e di Urgenza, Infermi Hospital Rimini, AUSL Romagna, Italy; ‡‡‡‡‡‡‡‡‡‡‡‡‡ General Surgery Service,Sanchinarro University Hospital, HM Hospitals Faculty of Health Sciences Camilo José Cela University Madrid, Spain; §§§§§§§§§§§§§ Department of Biotechnological and Applied Clinical Sciences, Division of General Surgery and Transplantation, University of L’Aquila, L’Aquila, Italy; ∥∥∥∥∥∥∥∥∥∥∥∥∥ Division of Hepatobiliary, Pancreatic and Transplantation Surgery, Polytechnic University of Marche, Ospedali Riuniti delle Marche, Ancona, Italy; ¶¶¶¶¶¶¶¶¶¶¶¶¶ Center for Advanced Treatment of Hepatobiliary and Pancreatic Diseases, Ageo Central General Hospital, Saitama, Japan; ############# Second Division of Surgery-Treviso-Department of Surgery, Oncology and Gastroenterology, DiSCOG, University of Padua, Padua, Italy; ************** Division of Surgical Oncology, University of Pittsburgh Medical Center, Pittsburgh, PA; †††††††††††††† Department of Surgery, Indiana University School of Medicine, Indianapolis, IN; ‡‡‡‡‡‡‡‡‡‡‡‡‡‡ Department of Surgery, HEBIPA-Hepatobiliary and Pancreatic Unit, Hospital de Santo António, Centro Hospitalar Universitário do Porto, Porto, Portugal; §§§§§§§§§§§§§§ Department HPB Surgery, Metropolitan Hospital, Athens, Greece; ∥∥∥∥∥∥∥∥∥∥∥∥∥∥ School of Medicine, Trinity College Dublin, Dublin, Ireland; ¶¶¶¶¶¶¶¶¶¶¶¶¶¶ Department of Surgery, Weill Cornell Medicine, New York-Presbyterian Hospital at Weill Cornell, New York, NY; ############## Department of Biomedical Sciences, Humanitas University, Milan, Italy; *************** Department of General Surgery, Division of General and Digestive Surgery, IRCCS Humanitas Research Hospital, Rozzano, Milan, Italy; ††††††††††††††† Department of Surgery, Donald and Barbara Zucker School of Medicine at Hofstra/Northwell, Manhasset, NY; ‡‡‡‡‡‡‡‡‡‡‡‡‡‡‡ Zucker School of Medicine at Hofstra, New Hyde Park, NY; §§§§§§§§§§§§§§§ Department of Clinical and Experimental Sciences, Surgical Clinic, University of Brescia, Brescia, Italy; ∥∥∥∥∥∥∥∥∥∥∥∥∥∥∥ Associazione per Donare la Vita Onlus, Pisa, Italy; ¶¶¶¶¶¶¶¶¶¶¶¶¶¶¶ Associazione Nastro Viola, Brescia, Italy; ############### Associazione Oncologica Pisana P. Trivella, Pisa, Italy; **************** Department of Surgery, John Hopkins University School of Medicine, Baltimore, MD; †††††††††††††††† Department of Surgery and Transplantation, University Hospital Zurich, University of Zurich, Zurich, Switzerland; ‡‡‡‡‡‡‡‡‡‡‡‡‡‡‡‡ Division of Hepatobiliary and Pancreas Surgery, Miami Cancer Institute, Miami, FL

**Keywords:** borderline-resectable pancreatic cancer, locally advanced pancreatic cancer, pancreatectomy with vascular resection, REDISCOVER Guidelines, REDISCOVER registry

## Abstract

**Objective:**

The REDISCOVER consensus conference aimed at developing and validating guidelines on the perioperative care of patients with borderline-resectable (BR-) and locally advanced (LA) pancreatic ductal adenocarcinoma (PDAC).

**Background:**

Coupled with improvements in chemotherapy and radiation, the contemporary approach to pancreatic surgery supports the resection of BR-PDAC and, to a lesser extent, LA-PDAC. Guidelines outlining the selection and perioperative care for these patients are lacking.

**Methods:**

The Scottish Intercollegiate Guidelines Network (SIGN) methodology was used to develop the REDISCOVER guidelines and create recommendations. The Delphi approach was used to reach a consensus (agreement ≥80%) among experts. Recommendations were approved after a debate and vote among international experts in pancreatic surgery and pancreatic cancer management. A Validation Committee used the AGREE II-GRS tool to assess the methodological quality of the guidelines. Moreover, an independent multidisciplinary advisory group revised the statements to ensure adherence to nonsurgical guidelines.

**Results:**

Overall, 34 recommendations were created targeting centralization, training, staging, patient selection for surgery, possibility of surgery in uncommon scenarios, timing of surgery, avoidance of vascular reconstruction, details of vascular resection/reconstruction, arterial divestment, frozen section histology of perivascular tissue, extent of lymphadenectomy, anticoagulation prophylaxis, and role of minimally invasive surgery. The level of evidence was however low for 29 of 34 clinical questions. Participants agreed that the most conducive means to promptly advance our understanding in this field is to establish an international registry addressing this patient population (https://rediscover.unipi.it/).

**Conclusions:**

The REDISCOVER guidelines provide clinical recommendations pertaining to pancreatectomy with vascular resection for patients with BR-PDAC and LA-PDAC, and serve as the basis of a new international registry for this patient population.

Pancreatic ductal adenocarcinoma (PDAC) remains an aggressive and frequently mortal malignancy.^1^ The poor prognosis of PDAC is influenced by late detection and poor response to existing oncologic treatments.^2–6^

In about one-third of the patients, PDAC exhibits a predominantly localized growth pattern.^7^ PDAC has the proclivity to surround and invade neighboring vascular structures and may be referred to as borderline-resectable (BR-PDAC) or locally advanced PDAC (LA-PDAC), based on the extent of involvement of these vessels.^8^ On practical grounds, a BR-PDAC is considered resectable to macroscopically negative margins. Resection of the portal vein and/or hepatic artery, however, may be required with the pathologic examination revealing a higher rate of microscopically positive resection margins, when compared to resectable PDAC. An LA-PDAC refers to an unresectable tumor. Resection of an LA-PDAC would typically require extensive retroperitoneal dissection or resection of an arterial segment and often vein resection, with no guarantee of complete tumor clearance. Historically, most patients with either BR-PDAC or LA-PDAC were not considered candidates for resection due to concerns of high morbidity and mortality, coupled with incomplete oncologic resection resulting in poor prognosis. Many considered such resection as a futile effort.^9,10^

The development of effective multiagent chemotherapy regimens has positively impacted the use of resection for patients with BR-PDAC and LA-PDAC. Indeed, the administration of chemotherapy in the neoadjuvant setting has become a game changer giving rise to the novel concept of “prognosis-based resectability” providing information about tumor biology and responsiveness.^11^ Following neoadjuvant therapy, PDAC is currently deemed resectable if there is no tumor progression or evidence of tumor regression, a decline of Ca 19.9 levels, and the general conditions of the patients are satisfactory. In an intention-to-treat analysis, neoadjuvant chemotherapy permitted resection in around 24% of patients with BR-PDAC and 9% with LA-PDAC.^12^ Therefore, this approach allows for a selection based on response to treatment. Oncology guidelines currently suggest considering surgical resection when such control or regression is observed.^8,13^

While this strategy based on “prognosis-based rationale” may justify a surgical approach to select patients with BR-PDAC and LA-PDAC, it adds new questions regarding the selection and management of these patients during the perioperative phase.^14–16^ The REDISCOVER international consensus conference was specifically organized to provide guidelines for clinical practice in this new context of decision-making based on oncologic responses, and still influenced by local institutional discussions at multidisciplinary tumor boards and surgical expertise.

## METHODS

The REDISCOVER guidelines were an initiative of the Italian Society of Surgery endorsed by the Pancreas Club Inc.

Four separate committees were formed. First, a 12-member Steering Committee was created based on clinical and scientific backgrounds, as well as an established surgical competence with BR-PDAC and LA-PDAC (Europe: 8, USA: 2, India: 1, South Australia: 1). The Steering Committee included the chairperson of the consensus conference (U.B.). This committee designated a Validation Committee consisting of 15 members (Europe: 12; USA: 2) chaired by a pancreatic surgeon familiar with the methodology (H.J.A.; USA) as well as 3 patient representatives and a Research Committee of 18 members (all from Europe) devoted to a comprehensive literature search for BR-PDAC and LA-PDAC. A large Expert Committee of 79 members (Europe: 64; USA: 7; Japan: 5; China: 3) was also created serving for the discussion at the consensus conference and the voting. Finally, a 19-member The REDISCOVER Multidisciplinary Advisory Board comprising members of medical and radiation oncology, radiology, nuclear medicine, diagnostic and interventional endoscopy, and pathology was selected to guarantee adherence to guidelines.

The methodology used to establish the REDISCOVER guidelines has been previously employed in other evidence-based guidelines.^17–20^ Briefly, working groups of experts and researchers used the Scottish Intercollegiate Guidelines Network (SIGN) methodology to evaluate the evidence and create guideline recommendations.^21^ The strength of recommendation was based on the GRADE rating.^22^ The Expert Committee then used the Delphi method to reach a consensus on the recommendations,^23^ and the Validation Committee used the AGREE II-GRS tool to assess the methodological quality of the guidelines and externally validate them.^24^ The Validation Committee operated autonomously since it was not involved in developing the recommendations and was not provided with any advance notice of the precise content of the guidelines before the meeting.

A total of 52 clinical questions were identified by the steering committee to be allocated to 5 working groups. Each working group consisted of 2 to 3 members of the steering committee, 1 to 2 senior researchers, and 2 to 3 junior researchers.

**FIGURE 1:**
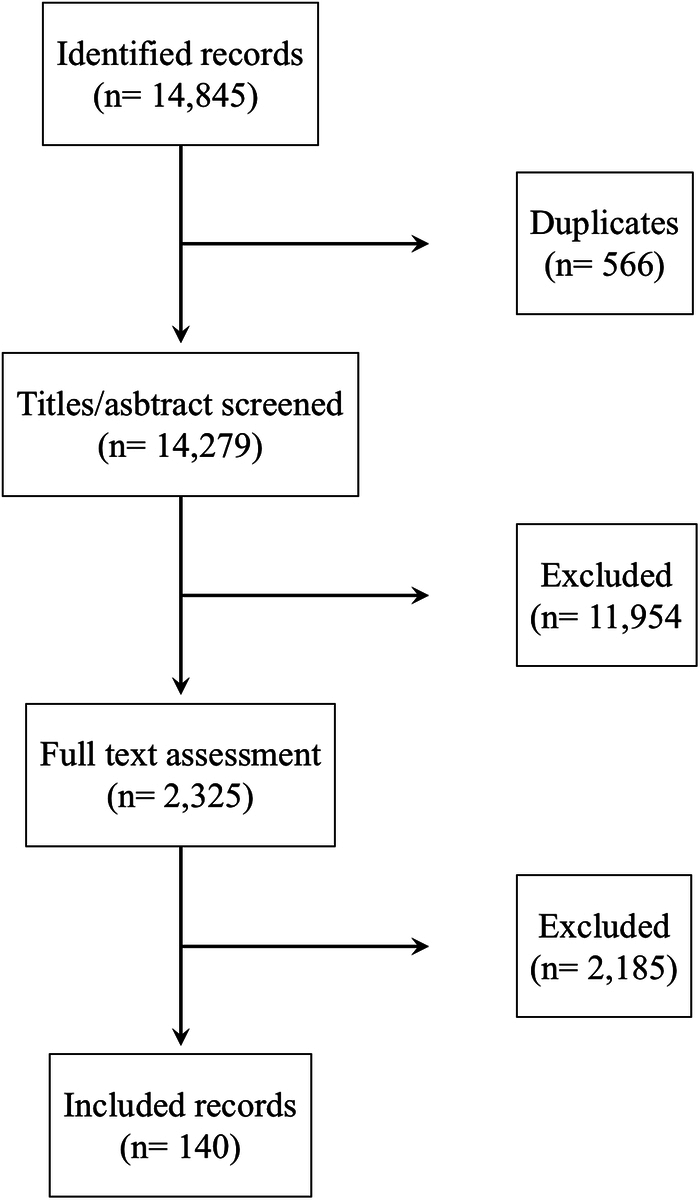
Flow chart of systematic literature review.

The working groups used the PubMed, Embase, and Cochrane databases to conduct systematic reviews of the literature for each question (the overall PRISMA flowchart is depicted in Fig. 1). Studies published in English that had a minimum sample size of 10 patients were included. Following screening, all studies deemed eligible were examined and condensed into distinct evidence tables.

The experts of working groups developed recommendations for each clinical question based on the quality of the evidence. A GRADE rating was attached to each recommendation. The Chairman received the final recommendations from each panel. Recommendations were combined into a questionnaire and distributed to the experts for the first online vote in accordance with the Delphi process. Experts could vote on whether they agreed or disagreed with the respective recommendations in addition to providing comments. For the first online Delphi session, an agreement rate of at least 90% was required. The recommendations that did not reach that agreement were sent back to the original working group for revision. A second online Delphi voting session was held with revised recommendations (agreement rate of at least 80%). The voting process was kept confidential and anonymous. The Chairman and researcher leaders were the only persons with access to the voting rounds’ results, which otherwise remained anonymous. All experts received the first and second online Delphi surveys on August 28 and September 10, 2023

An in-person meeting was held in Pisa, Italy, on September 17 and 18, 2023, during the 125th National Congress of the Italian Society of Surgery. Each working group delivered its evidence-based recommendations in a dedicated session. Following each statement, the audience used a digital voting system to indicate whether they agreed or disagreed with the proposed statement. To promote transparency and stimulate discussion, the results of the audience’s final vote were displayed in a real-time manner. The Validation Committee examined the recommendations’ wording and evaluated the methodology and quality of the guidelines for each topic according to the AGREE II-GRS tool. This was carried out following the presentation of the questions allocated to each working group during private Validation Committee sessions. The Validation Committee provided a report with the quality scores for every topic and recommendations for additions or deletions during the 2-day meeting. Recommendations, which had an initial audience approval percentage of <80% were revised/updated by the Validation Committee based on the discussions held by the experts in the audience and were then put to a second vote by the audience. The Chairman Committee and Expert Committee examined and approved all additional changes and recommendations.

## RESULTS

**FIGURE 2:**
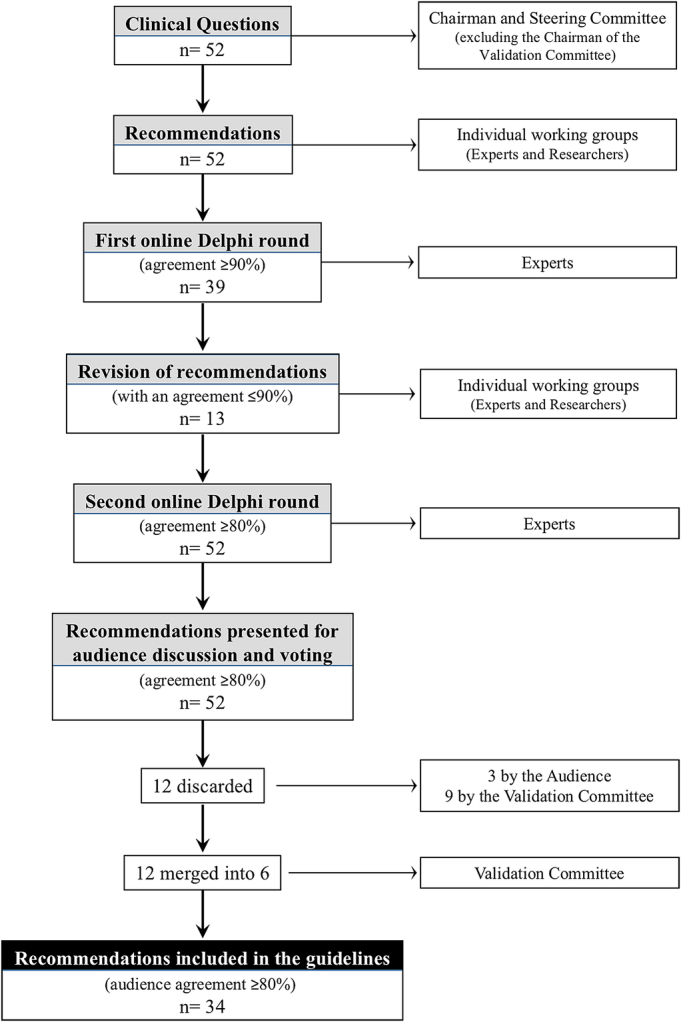
Flow chart of the guideline process.

While each recommendation was approved after the online Delphi rounds, minor phrasing modifications were made following the in-person meeting in Pisa, Italy. Twelve of the 52 clinical questions were consolidated into 6, 12 were dropped including 3 by the audience and 9 by the validation committee. Supplementary Table 1, Supplemental Digital Content 1, http://links.lww.com/SLA/F30 displays the 34 recommendations that were ultimately adopted. The clinical questions, accepted recommendations, audience agreement, expert agreement, grade of evidence, strength of recommendation, and quality score are listed in Supplementary Table 2, Supplemental Digital Content 1, http://links.lww.com/SLA/F30. Some comments are also added, when applicable. Figure 2 provides a flowchart of the process. A list of the clinical questions that were dropped is provided in Supplementary Table 3, Supplemental Digital Content 1, http://links.lww.com/SLA/F30. The consensus conference was attended by 136 participants from 18 countries.

**FIGURE 3:**
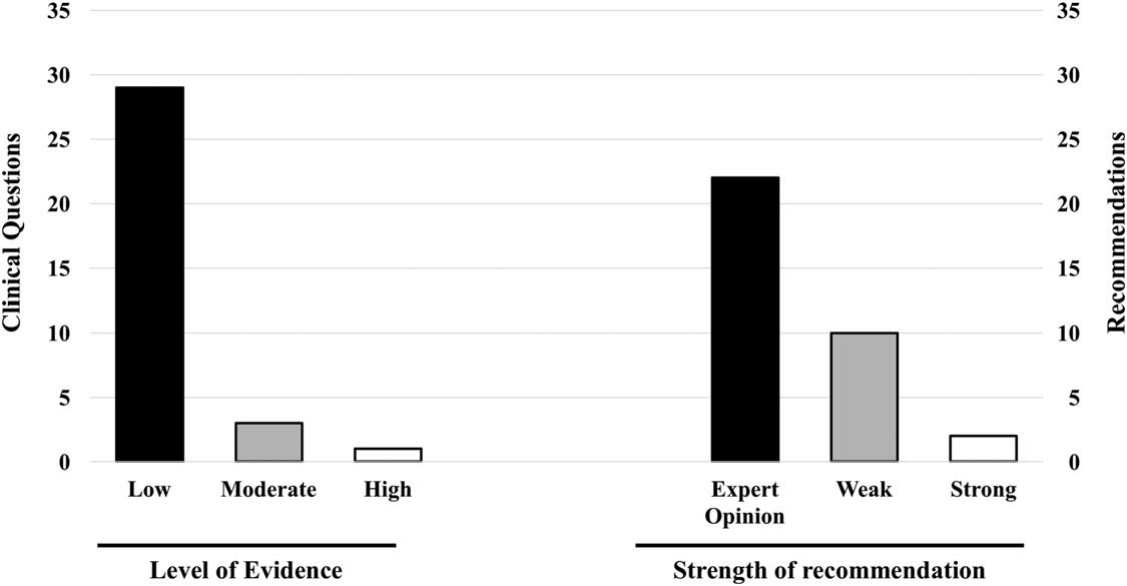
Histograms showing the level of evidence and the strength of recommendations.

Two recommendations were graded as “strong”—one of which was upgraded by experts—and 22 recommendations were graded as “expert opinion” because of the low level of evidence for 29 of the 34 clinical questions (85%) (Fig. 3). The 2 strong recommendations concern whether pancreatic resection should be pursued in patients with BR-PDAC after successful neoadjuvant treatments and whether epidural anesthesia should be preferred over standard anesthesia/analgesia. The low level of evidence was influenced by the many studies that reported BR and LA-PDAC as one unique entity.

The REDISCOVER guidelines outline specific recommendations for the present care of patients with BR-PDAC and LA-PDAC and indicate the several areas in which additional research is required.

Participants in the consensus meeting concluded that well-designed clinical trials and multi-institutional registries are urgently needed to improve the level of evidence and address several important issues about the treatment of BR-PDAC and LA-PDAC. Participants agreed that the most practical way to advance promptly our understanding is to establish an international registry, given that these studies may be challenging to conduct and may require much time to complete. The REDISCOVER registry is now available online (https://rediscover.unipi.it/).

## DISCUSSION

The REDISCOVER guidelines were developed to advance the understanding, management, and science around patients with BR-PDAC and LA-PDAC. Indeed, a growing number of patients with BR-PDAC and LA-PDAC are now considered eligible for surgery after receiving successful neoadjuvant therapies.^25–27^ An international assessment on the management of LA-PDAC among high-volume pancreatic surgeons revealed that all surgeons are willing to undertake portal vein resection in well-selected patients, and half of them were also willing to consider artery resection. Even in the case of oligometastatic liver metastases, around one-third of the experts would accept the option of resection. Nonetheless, this survey revealed a considerable variation in clinical practice, that is largely based on the lack of prospective studies.^28^ Therefore, it was clear that there is a great need for the international community of pancreas surgeons to convene and set some universal guidelines for evidence-based practice in these patients and determine areas where further evidence and collaboration are needed.

It is unrealistic to assume that the REDISCOVER guidelines could address all issues pertaining to the management and perioperative care of patients with LA-PDAC and BR-PDAC. Instead, they ought to be viewed as a first step toward an ongoing, worldwide cooperative endeavor to standardize these procedures. With this priority, we developed an online registry, which is currently available to enter cases on a large scale (https://rediscover.unipi.it/). It is expected that the international register REDISCOVER will serve as a tool for resolving some compelling issues. There is also a major need for high-quality prospective studies.

The REDISCOVER recommendations are not intended to supersede or conflict with already available oncology guidelines.^8,13^ Instead, they seek to address a number of surgical topics not covered in these documents and offer perspectives on a number of contentious issues pertaining to the use of oncology guidelines in surgical practice. In addition to that, some new concepts that were not included in earlier guidelines—such as the “test of time” and arterial divestment—need to be assessed in the REDISCOVER guidelines in light of the available evidence.

The REDISCOVER guidelines emphasize the importance of patient selection. Preoperative systemic therapy should be delivered to all patients with or without radiation. Surgery remains the treatment end-goal option for BR-PDAC and should be taken into consideration also in well-selected patients with LA-PDAC using stringent criteria including tumor regression/stability, a significant decline in Ca 19.9 levels, and limited to patients fit for surgery. Indeed, the new paradigm of prognosis-based resectability, emphasizing biological behavior over anatomic tumor features (ie, A-B-C approach), allows expert pancreatic surgeons to prepare for vessels and pancreatic resection.^11^ With this new strategy, surgeons must be always prepared to handle unplanned vein or artery resection and reconstruction during surgery.^29–31^

Vascular resections and reconstructions can be performed by liver transplant or vascular surgeons following preoperative planning or upon intraoperative consultation.^32,33^ Appreciating that timely support of vascular and liver transplant surgeons may not always be available has led the participants of the REDISCOVER consensus conference to advise that pancreatic surgeons should achieve proficiency and independence in vascular resection and reconstruction. Resection of BR-PDAC and, especially of LA-PDAC, requires the pancreatic surgeon to have the extra technical skill not usually encountered in routine pancreatic resections. The planning of the procedure based on imaging after neoadjuvant treatments,^34^ safe vascular control,^35^ portal hypertension management,^36^ preservation of blood supply to essential organs,^35^ workflow adaptation to patient’s anatomy,^35^ and patient management both before and after surgery^35^ are some of these specific technical challenges. Thus, a comprehensive reevaluation of the professional profile of pancreatic surgeons is necessary. Focused training in vascular techniques should be provided to the upcoming generation of pancreatic surgeons.

A substantial body of research suggests that outcomes of pancreatic resections improve if surgery is performed in high-volume centers.^37^ Although the postoperative mortality of pancreatic resections is improved when the historical threshold of 20 pancreatoduodenectomy procedures annually is applied, it is increasingly evident that this capped annual number of operations is only the start of a global quality improvement process.^38,39^ Furthermore, it was made evident during the REDISCOVER consensus meeting that not all large-volume centers agree on the oncologic value of arterial resections and/or are comfortable handling peripancreatic arteries. For this reason, the REDISCOVER guidelines introduced the idea of a center of excellence for pancreatic surgery. A center of excellence provides patients with comprehensive, interdisciplinary treatment delivered by highly skilled professionals, resulting in high-quality patient outcomes.^40^ Thus, this goes well beyond just volume, although high-volume (ie, >50–100 pancreatoduodenectomies/yr) is essential for this type of surgery. Recent benchmark studies demonstrated that centers operating on difficult cases offer better outcomes to all their patients, for example with lower rates of clinically relevant severe postoperative pancreatic fistula.^41–43^ One of the requirements for becoming a center of excellence should be to enroll patients in a prospective database or registry.

The annual incidence of pancreatic resections is approximately 6 per 10^5^ inhabitants.^44^ For BR-PDAC and LA-PDAC, the annual incidence drops to ~0.5 and 0.16 procedures per 10^5^ inhabitants, respectively.^45^ While these figures, clearly and further, support the need for BR-PDAC and LA-PDAC to be centralized for resection, it is important to note that centralization of pancreatic resections has only occurred in a few countries. Despite the overwhelming amount of data supporting this strategy, there are a number of obstacles that prevent centralization from being widely implemented.^46^

Arterial resection is still linked to significant death rates, even in high-volume centers with an established reputation in pancreatic surgery.^22,30,31^ Therefore, the REDISCOVER guidelines cannot generally advocate arterial resections in routine practice. Surgeons who are willing to pursue arterial resection must devote a significant amount of time and resources to learning how to perform it. The learning process is not just limited to surgical skills since a comprehensive preoperative assessment and planning are critical to the success of artery resection. Unplanned artery resection is associated with higher perioperative mortality than planned resection. Some unplanned arterial resections result from iatrogenic injury while peeling off the tumor from a visceral artery (also known as arterial divestment).^30,31^ Therefore, while arterial divestment may be a treatment option in selected patients to spare arterial resection,^47,48^ while accepting a non-negligible risk of false-negative frozen section histology potentially resulting in margin positive resection,^49^ surgeons must be prepared to unexpectedly proceed with arterial resection and reconstruction. Finally, up to 60% of the patients undergoing arterial resection did not receive neoadjuvant chemotherapy in the era of preoperative oncology treatments.^31^ Unanticipated arterial resection accounts for some of these pancreatectomies performed beyond the current guidelines, further underscoring the need for careful patient selection and inclusion in the registry. The REDISCOVER guidelines permit the prudent pursuit of arterial resections in highly selected patients (showing involvement of the celiac trunk and/or hepatic artery, but not of the superior mesenteric artery), operated upon by skilled pancreatic surgeons in centers of excellence, provided that a multidisciplinary tumor board decides to proceed with surgery and that the results are documented in a prospective database, and from now in the registry This is based on some pilot studies that demonstrate improved outcomes.^35,36,50,51^

Reviews of the literature and meeting discussions brought to light a few shortcomings in the BR-PDAC and LA-PDAC definitions as they stand. First, there is just 1 category of LA-PDAC (anatomic) compared to 3 categories of BR-PDAC (A-B-C: anatomic, biological, and conditional).^52,53^ Second, while encasement of both the celiac trunk and the superior mesenteric artery match the current definition of LA-PDAC, the REDISCOVER guidelines accept surgery as an option only when arterial involvement is limited to the celiac trunk. Third, there is a significant amount of heterogeneity in the interpretation of anatomic data.^28,54^ Moreover, tumor anatomy in cross-sectional imaging may not match tumor histology following neoadjuvant treatments, and may not be able to predict the extent of local malignant involvement.^55^ Fourth, in the current era of preoperative systemic therapy and multimodality management, the anatomic definition of BR-PDAC and LA-PDAC should be reassessed by the multispecialty board after neoadjuvant therapy to consider surgical resection or not. Such a decision must be individualized to each patient by the board. This decision should incorporate the patient’s response to neoadjuvant treatment, the patient’s age, and baseline conditions as well as integrate anatomic and biological criteria.

Finally, one important outcome of the REDISCOVER guidelines is the introduction of the concept of avoiding excessive delay in treatment initiation when a pathologic diagnosis has not been obtained after multiple attempts. In a selected group of patients who are well-informed and have an evident clinical and radiologic presentation for PDAC, starting neoadjuvant chemotherapy should be considered without the need for pretreatment tissue diagnosis. While the NCCN and ESMO guidelines both require tissue diagnosis before the administration of neoadjuvant treatments, they also recognize that, in cases where a multidisciplinary tumor board at a high-volume center agrees on the clinical diagnosis of PDAC and at least 2 biopsies failed to define a tissue diagnosis, oncology treatments may be initiated even lacking histology/cytology confirmation of PDAC.^8,13^

In conclusion, a group of experienced pancreas surgeons from all over the world came together at the REDISCOVER international consensus conference in an attempt to reach a consensus regarding the practical aspects of surgical therapy for patients with BR-PDAC and LA-PDAC. The REDISCOVER guidelines are only a starting point. The recommendations defined during the REDISCOVER international consensus conference should guide current pancreas surgeons and institutions on how to manage patients with BR-PDAC and LA-PDAC and guide future advances.

The very low level of evidence supporting the recommendations as well as the vibrant in-person discussion demonstrate how many aspects of perioperative care are still up to individual’s preference emphasizing the need for consensus and further development of evidence. The terms BR-PDAC and LA-PDAC are sometimes used interchangeably in the literature, and studies frequently incorporate data on both tumor phases, which added confusion to the topic. Perhaps, a new definition of BR-PDAC and LA-PDAC should be proposed that is less subjective in interpretation. As the development of high-quality evidence in this field will take a significant number of years, we hope that the implementation of the REDISCOVER international registry can supply some of the missing information.

## Supplementary Material
