# Supplementary material for: REDISCOVER International Guidelines on the Perioperative Care of Surgical Patients With Borderline-resectable and Locally Advanced Pancreatic Cancer
Source: Ann Surg. 2024 Feb 26;280(1):56–65. doi: 10.1097/SLA.0000000000006248 (PMC11161250; doi:10.1097/SLA.0000000000006248)
Supplement: Supplementary file 1 [file sla-280-056-s001.docx]

**Supplementary table 1.** Recommendations

|  |  | **Recommendations** | **LoE** | **SoR** |
| --- | --- | --- | --- | --- |
|  |  |  |  |  |
| **1** | **Centralization** | *There are no specific criteria to identify institutions for the centralization of BR and LA-PDAC, however, there is good evidence to support volume-outcome interaction in pancreatic surgery. Patients requiring pancreatectomy with vascular resection should be centralized to centers of excellence with specific experience in these procedures. Patients should be enrolled in prospective database and/or registries.* | Low | Expert Opinion |
|  |  |  |  |  |
| **2** | **Vascular resection** | *Vascular resection and reconstruction is a component of contemporary pancreatic surgery. Pancreatic surgeons should achieve proficiency in vascular resection and reconstruction.* | Low | Expert Opinion |
|  |  |  |  |  |
| **3** | **Staging of BR- and LA-PDAC** | *The clinical staging of patients with BR and LA PDAC should include pancreas protocol CT/MRI in addition to CT of the chest and baseline Ca19.9.* | Low | Expert Opinion |
|  |  |  |  |  |
| **4** | **Ca 19.9 non-secretors** | *In these patients baseline CEA and Ca 125 may be useful.* | Moderate | Weak |
|  |  |  |  |  |
| **5** | **FDG-PET for BR- and LA-PDAC** | *There is no specific role for routine FDG-PET in BR- and LA-PDAC. However, FDG-PET can be selectively employed in patients at higher risk of occult metastasis and to permit evaluation of metabolic response following preoperative oncology treatments.* | Low | Weak |
|  |  |  |  |  |
| **6** | **Endoscopic ultrasonography without biopsy** | *While most patients with BR- and LA-PDAC undergo preoperative EUS to achieve tissue cytology/histology, there is no evidence that EUS should be performed only for staging purposes.* | Low | Expert Opinion |
|  |  |  |  |  |
| **7** | **Pretreatment biopsy** | *Tissue diagnosis should be obtained in patients with BR- and LA-PDAC before preoperative oncology treatments. Inordinate delay of treatment should be avoided.* | Low | Expert Opinion |
|  |  |  |  |  |
| **8** | **Baseline staging laparoscopy** | *Baseline staging laparoscopy is not advised as a routine.* | Low | Expert Opinion |
|  |  |  |  |  |
| **9** | **Timing of surgery after neoadjuvant treatments** | *There is no evidence about the optimal timing for surgical resection in patients with BR- or LA-PDAC following neoadjuvant chemo ± radiation therapy. Following NCCN guidelines which indicate that surgery should be performed 4 to 8 weeks after completion of chemotherapy is recommended. All patients should should have their case discussed at multidisciplinary tumor board.* | Low | Expert Opinion |
|  |  |  |  |  |
| **10** | **Delaying surgery (“test of time”)** | *There is insufficient evidence to recommend waiting longer than 6 weeks after the end of neoadjuvant chemotherapy. Multidisciplinary tumor board discussion should recommend the best timing for surgical resection in individual patients.* | Low | Expert Opinion |
|  |  |  |  |  |
| **11** | **Molecular biomarkers in** **patient selection for surgery** | *There is currently no evidence of benefit from molecular biomarkers in patient selection for surgery. However genetic testing for inherited mutations and molecular tumor profiling is advised.* | Low | Weak |
|  |  |  |  |  |
| **12** | **Staging laparoscopy after neoadjuvant treatments (BR-PDAC)** | *In patients with BR-PDAC, staging laparoscopy may be recommended prior to pancreatic resection if there is suspicion of occult metastases or unresectability.* | Low | Weak |
|  |  |  |  |  |
| **13** | **Staging laparoscopy after neoadjuvant treatments (LA-PDAC)** | *In patients with LA-PDAC, staging laparoscopy is advised prior to laparotomy.* | Low | Weak |
|  |  |  |  |  |
| **14** | **Intraoperative ultrasounds** | *The assessment of resectability of BR-PDAC and LA-PDAC resectability following neoadjuvant therapies does not specifically call for the routine use of intraoperative ultrasound. Intraoperative ultrasound can be used to define anatomy.* | Low | Expert Opinion |
|  |  |  |  |  |
| **15** | **Pancreatic resection after neoadjuvant treatment (BR-PDAC)** | *In patients fit for surgery with BR-PDAC, surgical resection improves survival.* | Moderate | Strong* |
|  |  |  |  |  |
| **16** | **Pancreatic resection after neoadjuvant treatment (LA-PDAC)** | *In the absence of progression with good biological response complete surgical resection should be considered to improve survival. All patients should be discussed at a multidisciplinary tumor board. Only centers of excellence should perform these surgeries* | Low | Weak |
|  |  |  |  |  |
| **17** | **Pancreatic resection without neoadjuvant treatment (BR-PDAC)** | *In patients fit for surgery with BR-PDAC who, for any reason, cannot receive neoadjuvant multi-agent chemotherapy, surgery may improve survival. Neoadjuvant chemo-radiotherapy could be taken into consideration as an alternative to upfront surgery. All patients should be discussed at multidisciplinary tumor board. Only centers of excellence should perform these surgeries* | Low | Weak |
|  |  |  |  |  |
| **18** | **Pancreatic resection after neoadjuvant treatments and rising Ca 19.9 levels (BR-PDAC)** | *Rising Ca19.9 is considered a significant adverse prognostic factor for early recurrence after resection. All patients should be discussed at multidisciplinary tumor board. Only centers of excellence should perform these surgeries.* | Low | Expert Opinion |
|  |  |  |  |  |
| **19** | **Pancreatic resection after neoadjuvant treatments and oligometastic disease (BR-PDAC)** | 1. *Oligometastatic disease that develops during neoadjuvant therapy should be considered progression of disease and surgery should not be performed.* 2. *Patients with synchronous oligometastatic disease who receive neoadjuvant therapy and show a good response may be considered for surgical resection in very selected cases and after discussing with patient and family. All patients should be discussed at multidisciplinary tumor board. Only centers of excellence should perform these surgeries.* | Low | Expert Opinion |
|  |  |  |  |  |
| **20** | **Neoadjuvant chemo-radiation and postoperative complications** | *There is no evidence that chemo-radiation increases incidence and severity of postoperative complications compared to chemotherapy alone in patients with BR-PDAC undergoing pancreatic resection.* | Low | Weak |
|  |  |  |  |  |
| **21** | **Epidural anesthesia/analgesia** | *Epidural anesthesia can be used. There is no evidence of superiority over standard anesthesia/analgesia.* | High | Strong |
|  |  |  |  |  |
| **22** | **En-bloc resection of tumor and involved vessels** | *Attempting en-bloc resection is an established oncologic principle and should be followed.* | Low | Expert Opinion |
|  |  |  |  |  |
| **23** | **Grafts/patches for vascular reconstruction** | *Autologous grafts (either vessels or peritoneum), allografts (usually vessels), xenografts (usually bovine pericardium), and prosthetic grafts can all be used for vascular reconstruction at the time of pancreatectomy depending on availability, type of reconstruction, and surgeon preference.* | Moderate | Weak |
|  |  |  |  |  |
| **24** | **Frozen section of periarterial tissues** | *There is insufficient evidence to define the value of frozen section histology of periarterial tissues when discriminating between cancer invasion and perivascular fibrosis. Positive frozen section histology can be employed to decide to proceed with vascular resection or to abort the procedure.* | Low | Weak |
|  |  |  |  |  |
| **25** | **Arterial divestment** | *In BR-PDAC and LA-PDAC, there is no clear proof that arterial divestment increases R1 rates when compared to arterial resection.* | Low | Expert Opinion |
|  |  |  |  |  |
| **26** | **Lymphadenectomy** | *There is no evidence to support what an optimal lymphadenectomy is in BR-PDAC and LA-PDAC.* | Low | Expert Opinion |
|  |  |  |  |  |
| **27** | **Hepatic artery embolization in DPCAR** | *Embolization of the common* *hepatic artery, in preparation for distal pancreatectomy with en-bloc resection of the celiac trunk, does not completely prevent hepatic and/or gastric ischemia.* | High | Strong |
|  |  |  |  |  |
| **28** | **Hepatic artery reconstruction in DPCAR** | *The hepatic artery should be reconstructed when there are concerns of developing hepatic ischemia. However there is minimal evidence to define when the common hepatic artery should be reconstructed in a distal pancreatectomy with en-bloc resection of the celiac trunk.* | Low | Expert Opinion |
|  |  |  |  |  |
| **29** | **Gastric ischemia in DPCAR** | *In patients requiring total pancreatectomy with en-bloc resection of the celiac trunk gastric ischemia cannot be prevented in all patients. Surgeons should also be aware of venous congestion. If blood supply to the stomach appears sub-optimal, a low threshold to either partial or total gastrectomy should be adopted.* | Low | Expert Opinion |
|  |  |  |  |  |
| **30** | **Total pancreatectomy and artery resection** | *Total pancreatectomy is an option in selected patients, particularly when the risk of of pancreatic fistula is felt to be high. Surgeons performing arterial resection should register outcomes into prospective database and/or registries.* | Low | Expert Opinion |
|  |  |  |  |  |
| **31** | **Minimally invasive surgery in BR-PDAC** | *There is a role for minimally invasive pancreas resection in BR-PDAC. Further experience should continue in centers of excellence, meeting the criteria established by Miami and Brescia guidelines. Patients should be enrolled in prospective database and/or registries.* | Low | Expert Opinion |
|  |  |  |  |  |
| **32** | **Minimally invasive surgery in LA-PDAC** | *There is a very limited role for minimally invasive pancreas resection in LA-PDAC. Further experience should continue in centers of excellence, meeting the criteria established by Miami and Brescia guidelines. Patients should be enrolled in prospective database and/or registries* | Low | Expert Opinion |
|  |  |  |  |  |
| **33** | **Anticoagulation in vein resection** | *Data about anticoagulation management after pancreatectomy with vein resection and reconstruction is inconclusive.* | Low | Expert Opinion |
|  |  |  |  |  |
| **34** | **Anticoagulation in artery resection** | *Data about anticoagulation management after pancreatectomy with artery resection and reconstruction is sparse and inconclusive.* | Low | Expert Opinion |
|  |  |  |  |  |

**LoE**: Level of evidence; **SoR**: Strength of recommendation; *****Upgraded by experts; **DPCAR:** Distal pancreatectomy with resection of the celiac artery

**Supplementary table 2.** Clinical questions, recommendations, and relevant comments.

|  |  | **LoE** | **SoR** | **Expert agreement** | **Audience agreement** | **Quality Score** |  |
| --- | --- | --- | --- | --- | --- | --- | --- |
| **Q1** | **Are there criteria to identify institutions for centralization of the surgical management of patients affected by BR-PDAC and LA-PDAC?** |  |  |  |  |  |  |
| **R1** | *There are no specific criteria to identify institutions for the centralization of BR and LA-PDAC, however, volume-outcome interaction has been reported in pancreatic surgery. Patients requiring pancreatectomy with vascular resection should be centralized to centers of excellence with specific experience in these procedures. Patients should be enrolled in prospective database and/or registries.* | Low | Expert Opinion | 96% | 93% | 73% |  |
| **Comment** | The impact of centralization on improved outcomes following pancreatic surgery was propounded by Birkmeyer based on a reduced mortality in high-volume centres^1^. The main determinant of this improved outcome has been the ability to rescue patients who develop complications^2^. Given that surgery for borderline-resectable (BR-) and locally advanced (LA-) pancreatic ductal adenocarcinoma (PDAC) is more complex than that for standard pancreatoduodenectomy and fraught with the risk of increased morbidity and mortality^3^, the participants to the REDISCOVER consensus conference felt that despite the absence of high-level evidence, in the interest of patient safety and quality, such surgeries should be performed in centers meeting the highest quality standards, including specific experience with vascular resection and reconstruction during pancreatic resection (i.e. centers of excellence). | | | | | |  |
|  |  |  |  |  |  |  |  |
| **Q2** | **Should pancreatic surgeons receive specific training in vascular resection and reconstruction?** |  |  |  |  |  |  |
| **R2** | *Vascular resection and reconstruction is a component of contemporary pancreatic surgery. Pancreatic surgeons should achieve proficiency in vascular resection and reconstruction.* | Low | Expert Opinion | 95% | 97% | 73% |  |
| **Comment** | Globally, vascular resections and reconstructions are undertaken routinely by HPB surgeons involved in liver transplant^4^. There are some HPB centers where surgeons involve a vascular team when undertaking these surgeries^5^. Appreciating that timely support of liver transplant and vascular surgeons may not always be available, has led the participants to the REDISCOVER consensus conference to advise on the need for pancreatic surgeons to receive training in vascular resection and reconstruction. | | | | | |  |
|  |  |  |  |  |  |  |  |
| **Q3** | **Following neoadjuvant treatments, does surgery improves survival when compared to continued treatments in patients fit for surgery with BR-PDAC?** |  |  |  |  |  |  |
| **R3** | *In patients fit for surgery with BR-PDAC, surgical resection improves survival.* | Moderate | Strong* | 93% | 87% | 72% |  |
| **Comment** | Neoadjuvant treatments increase patient survival in BR-PDAC patients^6-8^. Resection improves survival in this patient population, according to the participants in the REDISCOVER consensus conference, despite the lack of a study comparing the outcomes of patients who are fit for surgery and receive either resection or ongoing oncology treatments. Probably, this study would be difficult to run, due to ethical concerns about sparing pancreatic resection in these patients. | | | | | |  |
|  |  |  |  |  |  |  |  |
| **Q4** | **Following neoadjuvant treatments, does surgical resection improves survival when compared to continued treatments in patients fit for surgery with LA-PDAC?** |  |  |  |  |  |  |
| **R4** | *In the absence of progression with good biological response complete surgical resection should be considered to improve survival. All patients should be discussed at a multidisciplinary tumor board. Only centers of excellence should perform these surgeries* | Low | Weak | 84% | 82% | 74% |  |
| **Comment** | Patients with LA-PDAC who respond well to neoadjuvant therapies may now consider pancreatic resection as a viable course of treatment^9^. Similar to BR-PDAC, no research has explicitly looked at whether surgery increases survival over ongoing cancer therapies. Once more, doing this study would be difficult. Since resection of LA-PDAC is associated with increased level of difficulty, possibly resulting in higher morbidity and mortality, these procedures should be performed only in centers meeting the highest quality standards, including specific experience with arterial resection and reconstruction during pancreatic resection (i.e. centers of excellence). | | | | | |  |
|  |  |  |  |  |  |  |  |
| **Q5** | **In patients fit for surgery with BR PDAC who, for any reason, cannot receive neoadjuvant chemotherapy, does surgery improves survival when compared to alternative treatments?** |  |  |  |  |  |  |
| **R5** | *In patients fit for surgery with BR-PDAC who, for any reason, cannot receive neoadjuvant multi-agent chemotherapy, surgery may improve survival. Neoadjuvant chemo-radiotherapy could be taken into consideration as an alternative to upfront surgery. All patients should be discussed at multidisciplinary tumor board. Only centers of excellence should perform these surgeries* | Low | Weak | 88% | 81% | 67% |  |
| **Comment** | The standard of care for BR-PDAC is neoadjuvant therapy. The majority of the survival benefit comes from multi-agent chemotherapy^10^. Thus, every patient should receive multi-agent neoadjuvant chemotherapy. Surgery is still an option for patients who are deemed suitable for it, even in cases where they are unable to get this treatment. | | | | | |  |
|  |  |  |  |  |  |  |  |
| **Q6** | **What is the best timing for surgical resection in patients with BR- or LA-PDAC after receiving neoadjuvant chemo ± radiation therapy?** |  |  |  |  |  |  |
| **R6** | *There is no evidence about the best timing for surgical resection in patients with BR- or LA-PDAC following neoadjuvant chemo +/- radiation therapy. Following NCCN guidelines which indicate that surgery should be performed 4 to 8 weeks after completion of chemotherapy is recommended. All patients should be discussed at multidisciplinary tumor board.* | Low | Expert Opinion | 98% | 97% | 74% |  |
| **Comment** | There is no conclusive evidence that waiting more than 6–8 weeks after neoadjuvant chemotherapy—that is, the amount of time needed to recover from the majority of toxicities associated with chemotherapy—improves the biological selection of surgical candidates (also known as the "test of time"). | | | | | |  |
|  |  |  |  |  |  |  |  |
| **Q7** | **In patients with BR-PDAC or LA-PDAC undergoing pancreatic resection, does neoadjuvant chemo-radiation increase incidence and severity of postoperative complications compared to chemotherapy alone?** |  |  |  |  |  |  |
| **R7** | *There is no evidence that chemo-radiation increases incidence and severity of postoperative complications compared to chemotherapy alone in patients with BR-PDAC undergoing pancreatic resection.* | Low | Weak | 96% | 95% | 80% |  |
| **Comment** | Actually, no study compared incidence and severity of postoperative complications in patients undergoing pancreatic resection for BR-PDAC based on type of neoadjuvant treatment. Studies providing surgical outcomes of BR-PDAC following chemo-radiation report “standard rates” of postoperative complications.  After stereotactic body radiation therapy in patients with BR- or LA-PDAC surgery delaying surgery >6 weeks can improve local disease control without increasing incidence and severity of postoperative complications^11^. | | | | | |  |
|  |  |  |  |  |  |  |  |
| **Q8** | **In patients with BR-PDAC who received neoadjuvant treatments, are fit for surgery, and have no evidence of distant metastasis but show rising Ca 19.9 levels does continued medical treatments improve survival when compared to pancreatic resection?** |  |  |  |  |  |  |
| **R8** | *Rising Ca19.9 is considered a significant adverse prognostic factor for early recurrence after resection. All patients should be discussed at multidisciplinary tumor board. Only centers of excellence should perform these surgeries.* | Low | Expert Opinion | 97% | 89% | 76% |  |
| **Comment** | Biological selection of surgical candidates following neoadjuvant treatments mostly relies on Ca 19.9 levels; nevertheless, certain patients who meet other selection criteria (such as good clinical conditions and absence of tumor development) do not respond to Ca 19.9 levels. After discussing options at the multidisciplinary tumor board, and possibly pursuing second-line therapy, surgery may be cautiously considered for these individuals. | | | | | |  |
|  |  |  |  |  |  |  |  |
| **Q9** | **In patients with BR-PDAC who received neoadjuvant medical treatments and are fit for surgery but have oligometastic disease, do continued medical treatments improve survival when compared to tumor resection?** |  |  |  |  |  |  |
| **R9** | 1. *Oligometastatic disease that develops during neoadjuvant therapy should be considered progression of disease and surgery should not be performed.* 2. *Patients with synchronous oligometastatic disease who receive neoadjuvant therapy and show a good response may be considered for surgical resection in very selected cases and after discussing with patient and family. All patients should be discussed at multidisciplinary tumor board. Only centers of excellence should perform these surgeries.* | Low | Expert Opinion | 91% | 92% | 69% |  |
| **Comment** | A potential intermediate disease stage called "oligometastatic" has been identified by an analysis of the outcomes of a subset of patients with metastatic PDAC. This stage is linked to prognostic implications that fall between those of localized and systemic illness^12^. In these patients, according to a position paper recently published by the Japan Pancreas Society and the International Association of Pancreatology, resection of the primary tumor combined with liver metastases may contribute to prolong survival^13^. This mostly applies to liver oligometastasis.  However, the concept of oligometastatic PDAC is not widely accepted. In addition, determining whether metastases are a small residual of a previously higher tumor burden (which could indicate a response to chemotherapy and possibly imply an indication for resection) or whether they have developed despite chemotherapy (which would indicate disease progression despite chemotherapy and contraindicate resection) is an additional and major challenge. | | | | | |  |
|  |  |  |  |  |  |  |  |
| **Q10** | **How BR- and LA-PDAC should be staged at the baseline?** |  |  |  |  |  |  |
| **R10** | *The clinical staging of patients with BR and LA PDAC should include pancreas protocol CT/MRI in addition to CT of the chest and baseline Ca19.9.* | Low | Expert Opinion | 92% | 94% | 69% |  |
| **Comment** | Baseline levels of serum Ca 19.9 should be obtained in the absence of cholestasis^14^. Additional predictive information can be obtained with CEA and Ca 125, particularly in patients who do not secrete Ca 19.9^15,16^. Magnetic resonance imaging of the liver can be selectively performed in patients with high Ca 19.9 levels or whenever liver metastases are suspected on contrast-enhanced computed tomography^17,18^. FDG-PET is not routine. It holds the potential to improve staging and could be used in selected cases^19^. | | | | | |  |
|  |  | | | | | |  |
| **Q11** | **Do Ca19.9 non-secretors with BR- and LA-PDAC need additional baseline studies?** |  |  |  |  |  |  |
| **R11** | *In these patients baseline CEA and Ca 125 can be useful.* | Moderate | Weak | 93% | 94% | 67% |  |
| **Comment** | Additional options include other biomarkers such as circulating tumor DNA, Dupan-2, and FDG-PET may also be used to define response to preoperative treatments^20-21^. | | | | | |  |
|  |  |  |  |  |  |  |  |
| **Q12** | **Is a biopsy always needed to start with preoperative oncology treatments in patients with BR- and LA-PDAC?** |  |  |  |  |  |  |
| **R12** | *Tissue diagnosis should be obtained in patients with BR- and LA-PDAC before preoperative oncology treatments. Inordinate delay of treatment should be avoided.* | Low | Expert Opinion | 96% | 99% | 74% |  |
| **Comment** | When determining whether a repeat tumor biopsy is necessary, a multidisciplinary tumor board should take into account factors such as a typical medical history, unambiguous radiological characteristics, and high levels of Ca 19.9 in the absence of cholestasis. Elevated Ca 19.9, along with high Ca 125 levels, further raises the risk of PDAC. The diagnosis of autoimmune pancreatitis must be ruled out. The patient should sign a specific informed consent form and be fully told about the lack of histology/cytology diagnosis when a timely tissue diagnosis is not possible. All things considered, the patient ought to begin treatment without undue delay (patient advocate). The 2023 edition of the PDAC guidelines from ESMO and NCCN both present this strategy^9,22^. | | | | | |  |
|  |  |  |  |  |  |  |  |
| **Q13** | **Is there a specific role for FDG-PET in BR- and LA-PDAC?** |  |  |  |  |  |  |
| **R13** | *There is no specific role for routine FDG-PET in BR- and LA-PDAC. However, FDG-PET can be selectively employed in patients at higher risk of occult metastasis and to permit evaluation of metabolic response following preoperative oncology treatments.* | Low | Weak | 97% | 97% | 76% |  |
| **Comment** | Selective use of FDG-PET may be considered in patients undergoing neoadjuvant therapy when other imaging modalities are inconclusive in assessing response to treatment and/or serum tumor markers are negative^19^. | | | | | |  |
|  |  |  |  |  |  |  |  |
| **Q14** | **Should all patients with BR- and LA-PDAC undergo preoperative endoscopic ultrasonography only for staging purposes?** |  |  |  |  |  |  |
| **R14** | *While most patients with BR- and LA-PDAC undergo preoperative EUS to achieve tissue cytology/histology, there is no evidence that EUS should be performed only for staging purposes.* | Low | Expert Opinion | 91% | 99% | 76% |  |
|  |  |  |  |  |  |  |  |
| **Q15** | **Should patients with BR-PDAC and LA-LAPC have a staging laparoscopy at the time of the diagnosis to determine if the following treatments can have a curative purpose?** |  |  |  |  |  |  |
| **R15** | *Baseline staging laparoscopy is not advised as a routine. Staging laparoscopy can detect occult metastases in selected patients.* | Low | Expert Opinion | 96% | 97% | 73% |  |
| **Comment** | Staging laparoscopy has higher diagnostic yields in patients with body/tail tumor location, larger tumor size, and elevated serum CA 19-9 and CEA^23,24^ | | | | | |  |
|  |  |  |  |  |  |  |  |
| **Q16** | **After neoadjuvant treatments, what is the role of staging laparoscopy**  **in patients with BR-PDAC?** |  |  |  |  |  |  |
| **R16** | *In patients with BR-PDAC, staging laparoscopy may be recommended prior to pancreatic resection if there is suspicion of occult metastases or unresectability.* | Low | Weak | 91% | 96% | 76% |  |
| **Comment** | Staging laparoscopy immediately before attempted resection of BR-PDAC is indicated if incidental diagnosis of oligometastasis is considered a contraindication to pancreatectomy. Patients with high tumor markers are at increased risk of harboring occult metastasis^25,26^ | | | | | |  |
|  |  |  |  |  |  |  |  |
| **Q17** | **After neoadjuvant treatments, what is the role of staging laparoscopy**  **in patients with LA-PDAC?** |  |  |  |  |  |  |
| **R17** | *In patients with LA-PDAC, staging laparoscopy is advised prior to laparotomy.* | Low | Weak | 91% | 90% | 75% |  |
| **Comment** | Staging laparoscopy immediately before attempted resection of LA-PDAC is indicated. Patients with high tumor markers are at increased risk of harboring occult metastasis^25,26^ | | | | | |  |
|  |  |  |  |  |  |  |  |
| **Q18** | **What is the role of molecular biomarkers in the selection of patients with BR- and LA-PDAC for surgery?** |  |  |  |  |  |  |
| **R18** | *There is no evidence of benefit of molecular biomarkers in patient selection for surgery. However genetic testing for inherited mutations and molecular tumor profiling is advised.* | Low | Weak | 99% | 98% | 69% |  |
| **Comment** | Molecular analysis has not yet entered routine clinical practice, adds costs that could not be covered by health insurance/system, and may prolong time to initiation of treatment. Therefore, routine molecular analysis cannot be currently recommended. | | | | | |  |
|  |  |  |  |  |  |  |  |
| **Q19** | **Is there an indication to wait longer than 6 week following completion of neoadjuvant/ primary oncology treatments (so called “test of time”) to ensure disease stability before surgery patients with BR and LA PDAC?** |  |  |  |  |  |  |
| **R19** | *There is insufficient evidence to recommend waiting longer than 6 weeks after the end of neoadjuvant chemotherapy. Multidisciplinary tumor discussion should recommend the best timing for surgical resection in each patient.* | Low | Expert Opinion | 99% | 94% | 62% |  |
| **Comment** | In patients with BR- and LA-PDAC, test-of-time is intended to ensure illness stability prior to surgery, however it may raise concerns on medical liability due to undue delay in delivery of surgery. The role of test of time in the management of these patients is still unclear. | | | | | |  |
|  |  |  |  |  |  |  |  |
| **Q20** | **After neoadjuvant treatments, is there a specific role for intraoperative ultrasound to improve assessment of resecability of BR-PDAC and LA-PDAC?** |  |  |  |  |  |  |
| **R20** | *The assessment of BR-PDAC and LA-PDAC resectability following neoadjuvant therapies does not specifically call for the routine use of intraoperative ultrasound. Intraoperative ultrasound can be used to define anatomy.* | Low | Expert Opinion | 97% | 94% | 69% |  |
|  |  |  |  |  |  |  |  |
| **Q21** | **Does epidural anesthesia/analgesia improve early outcomes of patients undergoing pancreatectomy with vascular resection compared to standard anesthesia/analgesia?** |  |  |  |  |  |  |
| **R21** | *Epidural anesthesia can be used. There is no evidence of superiority over standard anesthesia/analgesia.* | High | Strong | 96% | 93% | 69% |  |
| **Comment** | A prospective randomized controlled trial showed that thoracic epidural analgesia, when compared to patient-controlled intravenous analgesia, did not increase postoperative complications in pancreatoduodenectomy patients, but it was linked to increased vasopressor use and higher weight gain by postoperative day 4^27^. The use of epidural anesthesia in patients undergoing pancreatectomy with vascular resection has not been specifically examined. Therefore, based on concerns about hypotension, the use of epidural anesthesia should be carefully considered especially in patients with LA-PDAC who may require complex vascular reconstructions. | | | | | |  |
|  |  |  |  |  |  |  |  |
| **Q22** | **Does en-bloc resection of tumor and involved vessel(s) provide advantages over sequential resection of tumor followed by vascular resection and reconstruction?** |  |  |  |  |  |  |
| **R22** | *Attempting en-bloc resection is an established oncologic principle and should be followed.* | Low | Expert Opinion | 92% | 91% | 63% |  |
| **Comment** | A more extensive resection is unlikely to increase survival once the tumor has been breached. Therefore, as long as resection aims at cure, it is not an option to detach the specimen from the vessels to technically simplify the vascular steps of the procedure. | | | | | |  |
|  |  |  |  |  |  |  |  |
| **Q23** | **Which grafts/patches can be used for vascular reconstruction at the time of pancreatectomy?** |  |  |  |  |  |  |
| **R23** | *Autologous grafts (either vessels or peritoneum), allografts (usually vessels), xenografts (usually bovine pericardium), and prosthetic grafts can all be used for vascular reconstruction at the time of pancreatectomy depending on availability, type of reconstruction, and surgeon preference.* | Moderate | Weak | 97% | 99% | 68% |  |
|  |  |  |  |  |  |  |  |
| **Q24** | **Following neoadjuvant oncology treatments, how accurate is frozen section histology of periarterial tissues in discriminating between cancer invasion and perivascular fibrosis?** |  |  |  |  |  |  |
| **R24** | *There is insufficient evidence to define the value of frozen section histology of periarterial tissues when discriminating between cancer invasion and perivascular fibrosis. Positive frozen section histology can be employed to decide to proceed with vascular resection or to abort the procedure.* | Low | Weak | 98% | 96% | 71% |  |
| **Comment** | The main shortcoming of frozen section histology of periarterial tissues is a high false negative rate of approximately 30%, possibly influenced by sampling errors. On the other hand, false positive results are extremely rare^28^ | | | | | |  |
|  |  |  |  |  |  |  |  |
| **Q25** | **In BR-PDAC and LA-PDAC does arterial divestment increases R1 rates when compared to arterial resection?** |  |  |  |  |  |  |
| **R25** | *In BR-PDAC and LA-PDAC, there is no clear proof that arterial divestment increases R1 rates when compared to arterial resection.* | Low | Expert Opinion | 96% | 93% | 62% |  |
| **Comment** | Arterial divestment should be considered when dissection can be safely performed in the sub-adventitial space and the dissection plane appears to be free from tumor (see also comment to R24). Therefore, it is not an alternative approach to arterial resection. Some patients may have both arterial divestment and artery resection. Further experience with arterial divestment is required to refine indications to this approach and provide fully reliable results. | | | | | |  |
|  |  |  |  |  |  |  |  |
| **Q26** | **How should anticoagulation be managed (either short- or long-term) in patients undergoing pancreatectomy with vein resection and reconstruction?** |  |  |  |  |  |  |
| **R26** | *Data about anticoagulation management after pancreatectomy with vein resection and reconstruction is inconclusive.* | Low | Expert Opinion | 99% | 100% | 64% |  |
| **Comment** | In patients undergoing pancreatectomy with vein resection advantages of increasing anticoagulation over standard postoperative prophylaxis for deep venous thrombosis should be carefully balanced against the risk of post-pancreatectomy hemorrhage, that often exceeds that of thrombosis. | | | | | |  |
|  |  |  |  |  |  |  |  |
| **Q27** | **How should anticoagulation be managed (either short- or long-term) in patients undergoing pancreatectomy with artery resection and reconstruction?** |  |  |  |  |  |  |
| **R27** | *Data about anticoagulation management after pancreatectomy with artery resection and reconstruction is sparse and inconclusive.* | Low | Expert Opinion | 97% | 100% | 65% |  |
| **Comment** | While pancreatectomy with artery resection and reconstruction may be associated with an increased risk of post-pancreatectomy hemorrhage, complications resulting from arterial occlusion are often severe and difficult to manage. In patients undergoing pancreatectomy with artery resection and reconstruction advantages of increasing anticoagulation over standard postoperative prophylaxis for deep venous thrombosis should be carefully balanced against the risk of visceral ischemia potentially complicating arterial occlusion. | | | | | |  |
|  |  |  |  |  |  |  |  |
| **Q28** | **Following neoadjuvant oncology treatments, what is the optimal lymphadenectomy in BR-PDAC and LA-PDAC?** |  |  |  |  |  |  |
| **R28** | *There is no evidence to support what an optimal lymphadenectomy is in BR-PDAC and LA-PDAC.* | Low | Expert Opinion | 95% | 97% | 64% |  |
| **Comment** | All studies on the value of lymphadenectomy were conducted in patients undergoing upfront pancreatic resection in the pre-neoadjuvant chemotherapy era^29-32^. Therefore there is no evidence to suggest which lymphadenectomy should ideally pursued in patients with either BR-PDAC or LA-PDAC, after neoadjuvant treatments. | | | | | |  |
|  |  |  |  |  |  |  |  |
| **Q29** | **When should the common hepatic artery be reconstructed in a distal pancreatectomy with en-bloc resection of the celiac trunk?** |  |  |  |  |  |  |
| **R29** | *The hepatic artery should be reconstructed when there are concerns of developing hepatic ischemia. However there is minimal evidence to define when the common hepatic artery should be reconstructed in a distal pancreatectomy with en-bloc resection of the celiac trunk.* | Low | Expert Opinion | 93% | 90% | 69% |  |
| **Comment** | When the left gastric artery is resected along with the celiac trunk, ischemic gastropathy is an additional consequence of insufficient collateral blood supply through the pancreatoduodenal arcades. Preoperative embolization of the common hepatic artery does not completely prevent ischemic complications of distal pancreatectomy with en-bloc resection of the celiac trunk (either hepatic or gastric). Therefore, when deciding to proceed with this procedure, surgeons must be prepared to reconstruct the hepatic artery^33,34^. | | | | | |  |
|  |  |  |  |  |  |  |  |
| **Q30** | **In patients scheduled for distal pancreatectomy with en-bloc resection of the celiac trunk, does per protocol embolization of the common hepatic artery reduces incidence and severity of ischemic complications when compared to intraoperative assessment of collateral circulation with possible reconstruction of the hepatic artery?** |  |  |  |  |  |  |
| **R30** | *Embolization of the common* *hepatic artery, in preparation for distal pancreatectomy with en-bloc resection of the celiac trunk, does not completely prevent hepatic and/or gastric ischemia.* | High | Strong | 93% | 90% | 69% |  |
| **Comment** | See comment to R29 | | | | | |  |
|  |  |  |  |  |  |  |  |
| **Q31** | **In patients requiring arterial resection and reconstruction, either alone or in combination with the superior mesenteric-portal vein, does total pancreatectomy improves postoperative outcomes when compared to partial pancreatectomy?** |  |  |  |  |  |  |
| **R31** | *Total pancreatectomy is an option in selected patients, particularly when the risk of of pancreatic fistula is felt to be high. Surgeons performing arterial resection should register outcomes into prospective database and/or registries.* | Low | Expert Opinion | 95% | 90% | 69% |  |
| **Comment** | Arterial resection and reconstruction is feasible during partial pancreatectomy. However, total pancreatectomy may facilitate complex vascular reconstruction^4,35^. Consideration should be given to the effects of endocrine and exocrine insufficiency on quality of life when deciding whether to proceed with total pancreatectomy^36^. | | | | | |  |
|  |  |  |  |  |  |  |  |
| **Q32** | **In patients requiring total pancreatectomy with en-bloc resection of the celiac trunk, can gastric ischemia be prevented?** |  |  |  |  |  |  |
| **R32** | In patients requiring total pancreatectomy with en-bloc resection of the celiac trunk gastric ischemia cannot be prevented in all patients. Surgeons should also be aware of venous congestion. If blood supply to the stomach appears sub-optimal, a low threshold to either partial or total gastrectomy should be adopted. | Low | Expert Opinion | 96% | 95% | 60% |  |
| **Comment** | See comment to R29 | | | | | |  |
|  |  |  |  |  |  |  |  |
| **Q33** | **Is there a role for minimally invasive surgery in BR-PDAC?** |  |  |  |  |  |  |
| **R33** | *There is a role for minimally invasive pancreas resection in BR-PDAC. Further experience should continue in centers of excellence, meeting the criteria established by Miami and Brescia guidelines. Patients should be enrolled in prospective database and/or registries.* | Low | Expert Opinion | 96% | 83% | 62% |  |
| **Comment** | There is a dearth of experience with minimally invasive pancreatic resection after neoadjuvant therapies. Robotic assistance appears to facilitate dissection and could improve vascular reconstruction in these patients^37^ | | | | | |  |
|  |  |  |  |  |  |  |  |
| **Q34** | **Is there a role for minimally invasive surgery in LA-PDAC?** |  |  |  |  |  |  |
| **R34** | *There is a very limited role for minimally invasive pancreas resection in LA-PDAC. Further experience should continue in centers of excellence, meeting the criteria established by Miami and Brescia guidelines. Patients should be enrolled in prospective database and/or registries* | Low | Expert Opinion | 92% | 80% | 58% |  |
| **Comment** | See comment to R33 | | | | | |  |
|  |  |  |  |  |  |  |  |

**Q**: Clinical question; **R**: recommendation; **LoE**: Level of evidence; **SoR**: Strength of recommendation

***** Upgraded by experts

**REFERENCES**

1. Birkmeyer JD, Finlayson SR, Tosteson AN, Sharp SM, Warshaw AL, Fisher ES. Effect of hospital volume on in-hospital mortality with pancreaticoduodenectomy. *Surgery.* 1999;125:250-256.
2. Ghaferi AA, Birkmeyer JD, Dimick JB. Complications, failure to rescue, and mortality with major inpatient surgery in medicare patients. *Ann Surg.* 2009;250:1029-1034. doi: 10.1097/sla.0b013e3181bef697.
3. Mihaljevic AL, Hackert T, Loos M, et al. Not all Whipple procedures are equal: Proposal for a classification of pancreatoduodenectomies. *Surgery.* 2021;169:1456-1462. doi: 10.1016/j.surg.2020.11.030.
4. Boggi U, Napoli N, Kauffmann EF, et al. Pancreatectomy with resection and reconstruction of the superior mesenteric artery. *Br J Surg.* 2023;110:901-904. doi: 10.1093/bjs/znac363.
5. Sgroi MD, Narayan RR, Lane JS, et al. Vascular reconstruction plays an important role in the treatment of pancreatic adenocarcinoma. *J Vasc Surg.* 2015;61:475-80. doi: 10.1016/j.jvs.2014.09.003.
6. Ghaneh P, Palmer D, Cicconi S, et al. Immediate surgery compared with short-course neoadjuvant gemcitabine plus capecitabine, FOLFIRINOX, or chemoradiotherapy in patients with borderline resectable pancreatic cancer (ESPAC5): a four-arm, multicentre, randomised, phase 2 trial. *Lancet Gastroenterol Hepatol.* 2023;8:157-168. doi: 10.1016/S2468-1253(22)00348-X.
7. Versteijne E, van Dam JL, Suker M, et al. Neoadjuvant chemoradiotherapy versus upfront surgery for resectable and borderline resectable pancreatic cancer: Long-term results of the dutch randomized PREOPANC trial. *J Clin Oncol.* 2022;40:1220-1230. doi: 10.1200/JCO.21.02233.
8. Katz MHG, Shi Q, Meyers J, et al. Efficacy of Preoperative mFOLFIRINOX vs mFOLFIRINOX plus hypofractionated radiotherapy for borderline resectable adenocarcinoma of the pancreas: The A021501 phase 2 randomized clinical trial. *JAMA Oncol*. 2022;8:1263-1270. doi: 10.1001/jamaoncol.2022.2319.
9. National Comprehensive Cancer Network (NCCN) guidelines for pancreatic adenocarcinoma. Version 2.2023 – June 19, 2023. Accessed online on October 22, 2023 at <https://www.nccn.org/professionals/physician_gls/pdf/pancreatic.pdf>
10. Sugawara T, Rodriguez Franco S, Sherman S, et al. Neoadjuvant chemotherapy versus upfront surgery for resectable pancreatic adenocarcinoma: an updated nationwide study. *Ann Surg.* 2023 May 25. doi: 10.1097/SLA.0000000000005925.
11. Lin T, Reddy A, Hill C, et al. The timing of surgery following stereotactic body radiation therapy impacts local control for borderline resectable or locally advanced pancreatic cancer. *Cancers (Basel).* 2023;15:1252. doi: 10.3390/cancers15041252.
12. Frountzas M, Schizas D, Kykalos S, Toutouzas KG. "Oligometastatic pancreatic cancer" definition: The first step. *Hepatobiliary Pancreat Dis Int.* 2022;S:1499-3872(22)00153-9. doi: 10.1016/j.hbpd.2022.07.002.
13. Hashimoto D, Satoi S, Fujii T, et al. Is surgical resection justified for pancreatic ductal adenocarcinoma with distant abdominal organ metastasis? A position paper by experts in pancreatic surgery at the Joint Meeting of the International Association of Pancreatology (IAP) & the Japan Pancreas Society (JPS) 2022 in Kyoto. *Pancreatology.* 2023;23:682-688. doi: 10.1016/j.pan.2023.07.005.
14. Tsen A, Barbara M, Rosenkranz L. Dilemma of elevated CA 19-9 in biliary pathology. *Pancreatology.* 2018;18:862-867. doi: 10.1016/j.pan.2018.09.004.
15. Napoli N, Kauffmann EF, Ginesini M, et al. Ca 125 is an independent prognostic marker in resected pancreatic cancer of the head of the pancreas. *Updates Surg*. 2023;75:1481-1496. doi: 10.1007/s13304-023-01587-4.
16. Luo G, Liu C, Guo M, et al. Potential biomarkers in Lewis negative patients with pancreatic cancer. *Ann Surg.* 2017;265:800-805. doi: 10.1097/SLA.0000000000001741.
17. Alabousi M, McInnes MD, Salameh JP, et al. MRI vs. CT for the detection of liver metastases in patients with pancreatic carcinoma: a comparative diagnostic test accuracy systematic review and meta-analysis. *J Magn Reson Imaging.* 2021;53:38-48. doi: 10.1002/jmri.27056.
18. Kim HW, Lee JC, Paik KH, et al. Adjunctive role of preoperative liver magnetic resonance imaging for potentially resectable pancreatic cancer. *Surgery.* 2017;161:1579-1587. doi: 10.1016/j.surg.2016.12.038.
19. Abdelrahman AM, Goenka AH, Alva-Ruiz R, et al. FDG-PET Predicts neoadjuvant therapy response and survival in borderline resectable/locally advanced pancreatic adenocarcinoma. *J Natl Compr Canc Netw.* 2022;20:1023-1032.e3. doi: 10.6004/jnccn.2022.7041.
20. Kitahata Y, Kawai M, Hirono S, et al. Circulating tumor dna as a potential prognostic marker in patients with borderline-resectable pancreatic cancer undergoing neoadjuvant chemotherapy followed by pancreatectomy. *Ann Surg Oncol.* 2022;29(3):1596-1605. doi: 10.1245/s10434-021-10985-0.
21. Omiya K, Oba A, Inoue Y, et al. Serum DUPAN-2 could be an alternative biological marker for ca19-9 non-secretors with pancreatic cancer. *Ann Surg.* 2022 Jan 25. doi: 10.1097/SLA.0000000000005395.
22. Conroy T, Pfeiffer P, Vilgrain V, et al. Pancreatic cancer: ESMO clinical practice guideline for diagnosis, treatment and follow-up. *Ann Oncol.* 2023;S0923-7534(23)00824-4. doi: 10.1016/j.annonc.2023.08.009
23. Suker M, Koerkamp BG, Coene PP, et al. Yield of staging laparoscopy before treatment of locally advanced pancreatic cancer to detect occult metastases. *Eur J Surg Oncol.* 2019 Oct;45(10):1906-1911. doi: 10.1016/j.ejso.2019.06.004.
24. Gudmundsdottir H, Yonkus JA, Alva-Ruiz R, et al. Yield of staging laparoscopy for pancreatic cancer in the modern era: analysis of more than 1,000 consecutive patients. *J Am Coll Surg.* 2023;237:49-57.
25. van Dongen JC, Versteijne E, Bonsing BA, et al. The yield of staging laparoscopy for resectable and borderline resectable pancreatic cancer in the PREOPANC randomized controlled trial. *Eur J Surg Oncol.* 2023;49(4):811-817. doi: 10.1016/j.ejso.2022.12.011.
26. Satoi S, Yanagimoto H, Toyokawa H, et al. Selective use of staging laparoscopy based on carbohydrate antigen 19-9 level and tumor size in patients with radiographically defined potentially or borderline resectable pancreatic cancer. *Pancreas.* 2011;40:426-432. doi: 10.1097/MPA.0b013e3182056b1c.
27. Klotz R, Larmann J, Klose C, et al. Gastrointestinal complications after pancreatoduodenectomy with epidural vs patient-controlled intravenous analgesia: a randomized clinical trial. *JAMA Surg.* 2020;155:e200794. doi: 10.1001/jamasurg.2020.0794.
28. Nelson DW, Blanchard TH, Causey MW, Homann JF, Brown TA. Examining the accuracy and clinical usefulness of intraoperative frozen section analysis in the management of pancreatic lesions. *Am J Surg.* 2013;205(5):613-7; discussion 617. doi: 10.1016/j.amjsurg.2013.01.015.
29. Pedrazzoli S, DiCarlo V, Dionigi R, Mosca F, Pederzoli P, Pasquali C, Klöppel G, Dhaene K, Michelassi F. Standard versus extended lymphadenectomy associated with pancreatoduodenectomy in the surgical treatment of adenocarcinoma of the head of the pancreas: a multicenter, prospective, randomized study. Lymphadenectomy Study Group. *Ann Surg.* 1998;228:508-517. doi: 10.1097/00000658-199810000-00007.
30. Yeo CJ, Cameron JL, Lillemoe KD, Sohn TA, Campbell KA, Sauter PK, Coleman J, Abrams RA, Hruban RH. Pancreaticoduodenectomy with or without distal gastrectomy and extended retroperitoneal lymphadenectomy for periampullary adenocarcinoma, part 2: randomized controlled trial evaluating survival, morbidity, and mortality. *Ann Surg.* 2002;236(3):355-366; discussion 366-368. doi: 10.1097/00000658-200209000-00012.
31. Jang JY, Kang MJ, Heo JS, Choi SH, Choi DW, Park SJ, Han SS, Yoon DS, Yu HC, Kang KJ, Kim SG, Kim SW. A prospective randomized controlled study comparing outcomes of standard resection and extended resection, including dissection of the nerve plexus and various lymph nodes, in patients with pancreatic head cancer. *Ann Surg.* 2014;259:656-664. doi: 10.1097/SLA.0000000000000384.
32. Farnell MB, Pearson RK, Sarr MG, DiMagno EP, Burgart LJ, Dahl TR, Foster N, Sargent DJ; Pancreas Cancer Working Group. A prospective randomized trial comparing standard pancreatoduodenectomy with pancreatoduodenectomy with extended lymphadenectomy in resectable pancreatic head adenocarcinoma. *Surgery.* 2005;138:618-628; discussion 628-630. doi: 10.1016/j.surg.2005.06.044.
33. Nakamura T, Okada KI, Ohtsuka M, et al. Insights from managing clinical issues in distal pancreatectomy with en bloc coeliac axis resection: experiences from 626 patients. *Br J Surg.* 2023;110:1387-1394. doi: 10.1093/bjs/znad212.
34. Ueda A, Sakai N, Yoshitomi H, et al. Is hepatic artery coil embolization useful in distal pancreatectomy with en bloc celiac axis resection for locally advanced pancreatic cancer? *World J Surg Oncol.* 2019;17:124. doi: 10.1186/s12957-019-1667-8.
35. Napoli N, Kauffmann EF, Lombardo C, et al. Postoperative results, learning curve, and outcomes of pancreatectomy with arterial resection: a single-center retrospective cohort study on 236 procedures. *Int J Surg.* 2023 Dec 11. doi: 10.1097/JS9.0000000000000971.
36. Scholten L, Stoop TF, Del Chiaro M, Busch OR, van Eijck C, Molenaar IQ, de Vries JH, Besselink MG; Dutch Pancreatic Cancer Group. Systematic review of functional outcome and quality of life after total pancreatectomy. *Br J Surg.* 2019;106:1735-1746. doi: 10.1002/bjs.11296.
37. Robertson FP, Parks RW. A review of the current evidence for the role of minimally invasive pancreatic surgery following neo-adjuvant chemotherapy. *Laparoscopic, Endoscopic and Robotic Surgery* 2022; 5: 47-51. <https://doi.org/10.1016/j.lers.2022.03.003>.

**Supplementary table 3.** Discarded clinical questions and recommendations.

|  | **LoE** | **SoR** | **Expert agreement** | **Audience agreement** | |
| --- | --- | --- | --- | --- | --- |
|  |  |  |  |  | |
| **DISCARDED AFTER AUDIENCE DISCUSSIONS AND VOTING** | | | | |  |
|  | | | | |  |
|  |  |  |  |  | |
| **Following neoadjuvant treatments, in patients with radiologic encasement of the superior mesenteric artery does tumor resection improve survival when compared to continued medical treatments?** |  |  |  |  | |
| *In the absence of progression with good biological response complete surgical resection should be considered to improve survival. All patients should be discussed at MDT board. Only high-volume centers should perform these surgeries.* | Low | Expert Opinion | 88.1% | 72% | |
|  |  |  |  |  | |
| **In patients fit for surgery with non-metastatic LA-PDAC involving the superior mesenteric artery who, for any reason, cannot receive preoperative multi-agent chemotherapy, does surgery improve survival when compared to alternative treatments?** |  |  |  |  | |
| *In patients with non-metastatic LA-PDAC involving the superior mesenteric artery who are fit for surgery but, for any reason, are unable to receive preoperative multi-agent chemotherapy, chemo-radiotherapy should be considered as an alternative to upfront surgery. Given the high level of complexity involved in these procedures, upfront surgery should generally be avoided in these patients. If R2 resection may be avoided, pancreatectomy with resection and reconstruction of the superior mesenteric artery may be carefully evaluated in centers with specific experience and positive postoperative outcomes.* | Low | Weak | 80.2% | 55% | |
|  |  |  |  |  | |
| **In patients with LA PDAC who received neoadjuvant medical treatments and are fit for surgery but have oligometastic disease, do continued medical treatments improve survival when compared to tumor resection?** |  |  |  |  | |
| *In patients with LA-PDAC who received neoadjuvant medical treatments and are fit for surgery but have oligometastic disease, there is no evidence that resection improves survival when compared continued medical treatments. The best approach to oligometastasis in PDAC is determined by a variety of factors, including oncology and patient characteristics. In some patients with oligometastasis who responded to multi-agent chemotherapy, preliminary data suggest that tumor resection may be beneficial, particularly when tumor markers showed a clear decline, patients were in good clinical condition, and resection of the primary tumor aimed to local radicality. The option of resection should be carefully discussed in a multidisciplinary tumor board considering also the burden of surgery, candidly presented to patients, and documented in a written informed consent. Patients should be closely monitored, and outcome information should be entered into prospective databases.* | Low | Weak | 85.1% | 67% | |
|  |  |  |  |  | |
| **DISCARDED BY THE VALIDATION COMMITTEE** | | | | |  |
|  | | | | |  |
| **In patients fit for surgery with non-metastatic LA-PDAC involving the celiac trunk who, for any reason, cannot receive preoperative multi-agent chemotherapy, does surgery improve survival when compared to alternative treatments?** | Low | Weak | 86.1% | 81% | |
| *Chemo-radiotherapy should be taken into consideration instead of upfront surgery in patients with non-metastatic LA-PDAC involving the celiac trunk who are fit for surgery but, for any reason, are unable to receive preoperative multi-agent chemotherapy. In high volume centers, upfront surgery may be carefully considered if R2 resection can be avoided.* |  |  |  |  | |
|  |  |  |  |  | |
| **What is the best timing for of surgery surgical resection in patients with BR- or LA-PDAC who received primary/neoadjuvant chemo-radiation?** |  |  |  |  | |
| *There is no clear evidence about the best timing of surgery in patients with BR- or LA-PDAC following primary/neoadjuvant chemo-radiation. However, delaying surgery >10 or >20 weeks, while adding a short course of additional chemotherapy, can improve pathologic response.* | Low | Weak | 81.2% | 80% | |
|  |  |  |  |  | |
| **Is there an ideal number of chemotherapy cycles before surgery?** |  |  |  |  | |
| *There is no clear evidence about the ideal number of chemotherapy cycles before surgery. While more preoperative chemotherapy cycles could prolong survival, the decision when chemotherapy is completed and the patient can be considered for surgery, should be taken on an individual basis by a multidisciplinary pancreas tumor board.* | Low | Weak | 97% | 95% | |
|  |  |  |  |  | |
| **In patients with BR-PDAC undergoing pancreatic resection, does neoadjuvant chemo-radiation improve oncologic outcomes compared to chemotherapy alone?** |  |  |  |  | |
| *Chemo-radiation does not appear to improve oncologic outcomes of patients with BR-PDAC undergoing pancreatic resection, despite higher rates of R0 resection and improved pathological response.* | High | Strong | 91.1% | 92% | |
|  |  |  |  |  | |
| **In patients with LA-PDAC, does primary chemo-radiation improve oncologic outcomes when compared to chemotherapy alone?** |  |  |  |  | |
| *Currently available data do not fully support the hypothesis that chemo-radiation improves oncologic outcomes of LA-PDAC when compared to chemotherapy alone. Well-designed randomized control trials are required to answer this question.* | Low | Weak | 92.1% | 91% | |
|  |  |  |  |  | |
| **In patients with BR-PDAC who are fit for surgery, do ablation therapies improve oncologic outcomes compared to pancreatic resection?** |  |  |  |  | |
| *No study has compared ablation therapies to surgery in patients with BR-PDAC fit for surgery. Therefore, at the present time, there is no evidence supporting the hypothesis that ablation therapies could improve oncologic outcomes compared to pancreatic resection.* | Low | Weak | 92.1% | 98% | |
|  |  |  |  |  | |
| **In patients with LA-PDAC who are fit for surgery, do ablation therapies improve oncologic outcomes compared to pancreatic resection?** |  |  |  |  | |
| *Currently available studies have a retrospective design and are at high risk of selection bias. Therefore, there is no evidence that ablation therapies can improve oncologic outcomes compared to pancreatic resection in patients with LA-PDAC. Preliminary data suggest that ablation therapies could be worth of further investigation.* | Low | Weak | 91.1% | 96% | |
|  |  |  |  |  | |
| **In patients with LA PDAC who received primary/neoadjuvant medical treatments, are fit for surgery, and have no evidence of distant metastasis but show rising Ca 19.9 levels do continued medical treatments improve survival when compared to tumor resection?** |  |  |  |  | |
| *There is no evidence that continued medical treatments improve survival when compared to tumor resection in patients with LA-PDAC who received neoadjuvant medical treatments, are fit for surgery, and have no evidence of distant metastasis but show rising Ca 19.9 levels. Response of Ca 19.9 to neoadjuvant medical treatments provides relevant prognostic information and is used to select surgical candidates. Probably because of this background, the literature does not provide specific information. Whether or not these patients could be offered resection (after chemotherapy switch), should be carefully defined in a multidisciplinary pancreatic tumor board. Potential advantages of pancreatic resection should be carefully balanced against predictably high postoperative morbidity and mortality rates.* | Low | Weak | 94.1% | 89% | |
|  |  |  |  |  | |
| ***In patients requiring resection and reconstruction of the celiac trunk/hepatic artery and the superior mesenteric artery, that typically includes also resection and reconstruction of the superior mesenteric-portal vein, does total pancreatectomy improves postoperative outcomes when compared to partial pancreatectomy?*** |  |  |  |  | |
| *Partial pancreatectomy is barely ever feasible in patients undergoing pancreatectomy with simultaneous resection and reconstruction of the celiac trunk/hepatic artery and the superior mesenteric artery. In this specific setting, total pancreatectomy facilitates both venous and arterial reconstruction.* | Low | Weak | 96% | 88% | |
|  |  |  |  |  | |
| **MERGED WITH ANOTHER CLINICAL QUESTION BY THE VALIDATION COMMITTEE** | | | | |  |
|  | | | | |  |
| **In patients with LA-PDAC undergoing pancreatic resection, does primary chemo-radiation increase incidence and severity of postoperative complications compared to chemotherapy alone?** |  |  |  |  | |
| *There is no evidence that continued medical treatments improve survival when compared to tumor resection in patients with LA-PDAC who received neoadjuvant medical treatments, are fit for surgery, and have no evidence of distant metastasis but show rising Ca 19.9 levels. Response of Ca 19.9 to neoadjuvant medical treatments provides relevant prognostic information and is used to select surgical candidates. Probably because of this background, the literature does not provide specific information. Whether or not these patients could be offered resection (after chemotherapy switch), should be carefully defined in a multidisciplinary pancreatic tumor board. Potential advantages of pancreatic resection should be carefully balanced against predictably high postoperative morbidity and mortality rates.* | Low | Weak | 97% | 90% | |
|  |  |  |  |  | |
| ***Should patients with LA-PDAC undergo staging laparoscopy to determine intent of treatment?*** |  |  |  |  | |
| *Baseline staging laparoscopy is not advised as a routine in patients with LA-PDAC, but can be pursued in selected patients. Staging laparoscopy can add information about occult peritoneal and liver metastases.* | Low | Expert Opinion | 93.1% | 100% | |
|  |  |  |  |  | |
| ***After neoadjuvant treatments, is there a specific role for intraoperative ultrasound to improve assessment of resecability in LA-PDAC?*** |  |  |  |  | |
| *The assessment of LA-PDAC resectability following neoadjuvant therapies does not specifically call for the use of intraoperative ultrasound. Intraoperative ultrasound can be used to address particular problems, depending on the operator's skills.* | Low | Weak | 96% | 97% | |
|  |  |  |  |  | |
| ***In LA-PDAC does arterial divestment increases R1 rates when compared to arterial resection?*** |  |  |  |  | |
| *In LA-PDAC without clear arterial involvement, there is no proof that arterial divestment increases R1 rates when compared to arterial resection. In patients with overt or suspected arterial involvement insisting on arterial divestment is contraindicated. Arterial resection should instead be performed in these patients to maintain a curative intention.* | Low | Weak | 96% | 93% | |
|  |  |  |  |  | |
| ***Following primary oncology treatments, what is the ideal lymphadenectomy in LA-PDAC?*** |  |  |  |  | |
| *There is no evidence about the ideal lymphadenectomy in LA-PDAC. Prospective and randomized studies on level of lymphadenectomy in PDAC were run in the pre-chemotherapy era, and mostly in resectable PDAC. Due to the need to achieve clear vascular exposure and proceed to safe vascular reconstruction, lymphadenectomy pursued in LA-PDAC often matches the concept of extended lymphadenectomy, formerly established for upfront resection. The entire extrapancreatic nerve plexus is typically removed along with nodes in these patients.* | Low | Weak | 95% | 94% | |
|  |  |  |  |  | |
| ***In patients requiring resection and reconstruction of the superior mesenteric artery, either alone or in combination with the superior mesenteric-portal vein, does total pancreatectomy improves postoperative outcomes when compared to partial pancreatectomy?*** |  |  |  |  | |
| *Total pancreatectomy is an option in selected patients in whom the risk of pancreatic fistula is felt to be high. Surgeons performing arterial resection should register outcomes into prospective database and/or registries* | Low | Expert opinion | 95% | 85% | |

**LoE**: Level of evidence; **SoR**: Strength of recommendation
